# A Whole-Cortex Probabilistic Diffusion Tractography Connectome

**DOI:** 10.1523/ENEURO.0416-20.2020

**Published:** 2021-02-02

**Authors:** Burke Q. Rosen, Eric Halgren

**Affiliations:** 1 Neurosciences graduate program, University of California, San Diego, La Jolla, CA 92093; 2 Departments of Radiology and Neurosciences, University of California, San Diego, La Jolla, CA 92093

**Keywords:** diffusion MRI, human, Human Connectome Project, structural connectome, tractography

## Abstract

The WU-Minn Human Connectome Project (HCP) is a publicly-available dataset containing state-of-the-art structural magnetic resonance imaging (MRI), functional MRI (fMRI), and diffusion MRI (dMRI) for over a thousand healthy subjects. While the planned scope of the HCP included an anatomic connectome, resting-state fMRI (rs-fMRI) forms the bulk of the HCP’s current connectomic output. We address this by presenting a full-cortex connectome derived from probabilistic diffusion tractography and organized into the HCP-MMP1.0 atlas. Probabilistic methods and large sample sizes are preferable for whole-connectome mapping as they increase the fidelity of traced low-probability connections. We find that overall, connection strengths are lognormally distributed and decay exponentially with tract length, that connectivity reasonably matches macaque histologic tracing in homologous areas, that contralateral homologs and left-lateralized language areas are hyperconnected, and that hierarchical similarity influences connectivity. We compare the dMRI connectome to existing rs-fMRI and cortico-cortico-evoked potential connectivity matrices and find that it is more similar to the latter. This work helps fulfill the promise of the HCP and will make possible comparisons between the underlying structural connectome and functional connectomes of various modalities, brain states, and clinical conditions.

## Significance Statement

The tracts between cortical parcels can be estimated from diffusion magnetic resonance imaging (dMRI), but most studies concentrate on only the largest connections. Here, we present an atlas, the largest and most detailed of its kind, showing connections among all cortical parcels. Connectivity is relatively enhanced between frontotemporal language areas and homologous contralateral locations. We find that connectivity decays with fiber tract distance more slowly than predicted by brain volume and that structural and stimulation-derived connectivity are more similar to each other than to resting-state functional MRI (rs-fMRI) correlations. The connectome presented is publicly available and organized into a commonly used scheme for defining brain areas to enable ready comparison to other brain imaging datasets of various modalities.

## Introduction

In the 21st century, advances in computation, theory, and neuroimaging have spurred a broad and intense interest in the anatomic connections and physiological correlations among human brain areas. Bivariate functional connectivity has given way to full functional connectomes, the most comprehensive of which may be the WU-Minn Human Connectome Project (HCP)’s resting-state functional magnetic resonance imaging (rs-fMRI) dense connectome (Van Essen et al., 2013). The planned scope of WU-Minn HCP also included a full anatomic connectome (Van Essen and Ugurbil, 2017), and the project has collected, curated, and preprocessed diffusion MRI (dMRI) data for 1065 subjects. However, a structural connectome has to date not been released for these data. This report seeks to address this omission by presenting a full-cortex anatomic connectome derived from local, probabilistic tractography.

dMRI techniques detect white matter by registering the orientation biases of water molecule diffusion within myelinated axons. The majority of dMRI studies focus on differences in specific connections between treatment groups. In contrast, we seek here to present a robust, densely populated average connectivity matrix for the entire cortex using data from a large, healthy sample. Local dMRI fiber tract tracing algorithms can be broadly organized into two classes: deterministic, e.g., dsi-studio (Yeh et al., 2013), and probabilistic, e.g., probtrackX (Behrens et al., 2007). Deterministic tractography considers the most likely orientation at each voxel yielding the maximum likelihood tracts whereas probabilistic tractography considers the entire distribution of possible orientations, yielding a probability cloud of connections. As our goal is instead to explore all possible connections between regions, we employed local, probabilistic tractography (Behrens et al., 2007). This method has been validated against macaque retrograde tracers within-species (Donahue et al., 2016), and the dMRI protocol and equipment used for the WU-Minn HCP database were optimized in anticipation of this analysis (Sotiropoulos et al., 2013).

The physiological relevance of a connectome is maximized if its nodes form functionally distinct areas. Within the scope of cortex, this amounts to selecting a parcellation scheme. The HCP multimodal parcellation (HCP-MMP1.0; Glasser et al., 2016) has several advantages: its boundaries are both functionally and anatomically guided, it has sufficient parcels (360) to generate a rich connectome while few enough that the parcels’ extents comfortably exceed the dMRI voxel size, and mechanisms exist (Fischl et al., 2004) for it to be readily applied to individuals. Most importantly, the HCP-MMP1.0 parcellation is publicly available and widely adopted, facilitating the comparison of the generated matrices to other structural and functional connectomes.

Given the computational intensity of dMRI fiber tractography and the field’s inclination toward elucidating specific connections, it is not surprising that the number of existing publicly available dMRI datasets exceeds that of finished, readily applicable connectivity matrices. However, there do exist some prior examples. A brief overview of the advantages of this dMRI connectome compared to the USC Multimodal connectivity database (http://umcd.humanconnectomeproject.org) contains two dMRI tractography connectomes with standard surface-based parcellations: Hagmann (Hagmann et al., 2008) and ICBM (Mori et al., 2008), with sample-sizes of 5 and 138, respectively. A third is available at http://www.dutchconnectomelab.nl, which contains 114 controls. All of these use the Desikan–Killiany atlas (Desikan et al., 2006), which consists of 68 cortical parcels, and were produced with deterministic tractography. An atlas of major fiber tracts for the HCP 1200 cohort has recently released at http://brain.labsolver.org (Yeh et al., 2018). However, this deterministic tractography connectome is spatially coarse, consisting of only 54 cortical parcels, and lacks dynamic range and statistical dispersion, as weaker connections are unrepresented, rendering the connectivity matrix nearly binary. The HCP-MMP1.0 atlas employed here has more than five times as many parcels while retaining the functional distinctness of areas. In contrast to the relatively sparse existing deterministic matrices, the probabilistic approach may better resolve weak or low probability connections leading to densely populated connectivity matrices like those found non-human primate tracing studies (Markov et al., 2014). Furthermore, the cohort studied is large and many other types of data are available for the same individuals including the National Institutes of Health (NIH) neuropsychological toolbox (Gershon et al., 2013), as well as fMRI and magneto-encephalography (MEG) data for resting-state and cognitive tasks, permitting within-cohort comparison to functional connectivity. See Table 1 for a brief overview of the advantages of the dMRI connectome described here.

**Table 1:** Connectome features

Connectome Features
Probabilistic methodology sensitive to weak connections yielding a fully-populated, un-thresholded connectome
Cortex parcellated into the standardized, relatively dense, and functionally relevant HCP-MMP1.0 atlas
Large normative sample size (*N* = 1065)
Enables comparison with other measures in the WU-Minn HCP and other cohorts

The following report presents a novel structural connectome of the human neocortex based on probabilistic diffusion tractography. The connectome is partially validated against retrograde tracing in macaques and the relationship between tract length and connection strength is quantified. Further validation is provided by reasonable connectivity properties between contralateral homologous parcels, within language cortex, and between parcels lying at similar levels of the cortical hierarchy. Finally, the dMRI connectome is compared with cortico-cortico evoke potential (CCEP) and rs-fMRI derived connectivity.

## Materials and Methods

### Subjects and data sources

No new data were collected for this study, and the existing data used was gathered from publicly available databases. Individual subject’s high-resolution T1-weighted structural magnetic resonance volumes (MRI), diffusion images (dMRI), and group average grayordinate resting-state function MRI (rs-fMRI) connectivity were gathered from the HCP’s WU-Minn 1200 release (Van Essen et al., 2013) at https://db.humanconnectome.org. The diffusion imaging dataset consists of 1065 individuals (575 women), aged 22–36+ years old. The rs-fMRI group average cohort consists of 1003 individuals, 998 of whom are also in the dMRI dataset. These datasets include some twin and non-twin siblings. However, individuals’ family structure, as well as exact age, handedness, and ethnicity are access-restricted to protect the privacy of the subjects and these data were not requested as they are not critical to this study. Group-average dense T1w/T2w myelination index were gathered from the same source. Macaque retrograde tracer connectivity was sourced from Markov et al. (2014; their supplementary table 6). Parcel-by-parcel values were averaged across monkey and hemisphere. Group average, parcellated CCEP connectivity was gathered from the v1903 release of the Functional Brain Tractography project (F-TRACT; David et al., 2013; Trebaul et al., 2018) at https://f-tract.eu.

### Cortical parcellation and functional networks

The HCP multimodal parcellation scheme (HCP-MMP1.0), consisting of 180 cortical parcels per hemisphere, was projected from the Workbench (Marcus et al., 2011) 32k grayordinate template brain to the FreeSurfer (Fischl, 2012) ico5 fsaverage template as per (Coalson et al., 2016). Using the FreeSurfer reconstruction directories gathered from the database, surface-based fsaverage parcel labels were mapped onto each individual’s white matter surface using spherical landmark registration (fs_label2label; Fischl et al., 1999). Grayordinate rs-fMRI connectivity values were morphed to the ico5 fsaverage template then averaged within each parcel. Finally, individual’s surface-based parcel labels were converted to binary volumes marking the gray matter–white matter boundary (mri_label2vol) to serve as seed and target regions for probabilistic tractography. Workbench and FreeSurfer functions were sourced from releases 1.2.3 and 6.0, respectively.

To facilitate interpretation of the connectome, parcels were ordered and grouped into functional networks adapted from (Ji et al., 2019), which applied iterative Louvain clustering (Blondel et al., 2008; Rubinov and Sporns, 2010) and other criteria to a rs-fMRI connectivity. These functional groupings and parcel order were selected as they were also generated using (a subset of) the WU-Minn HCP dataset and the HCP-MMP1.0 parcellation scheme. For this study, the parcels of the left and right hemispheres were separated and the order and groupings of the left hemisphere in Ji et al. (2019) were used for homologous parcels in the both right and left hemisphere, respectively, when combining data across hemispheres. Two pairs of the original networks (primary and secondary visual, ventral, and posterior multimodal) contained too few parcels for effective analysis and were highly interrelated. These network pairs were simplified by combining them into visual and multimodal groups, yielding 10 functional networks per hemisphere (Table 2).

**Table 2:** Parcel order and network assignment

Idx.	Parcel	Orig.	Network	Idx.	Parcel	Orig.	Network	Idx.	Parcel	Orig.	Network
1	V1	1	Cingulo-opercular	61	46	84	Cingulo-opercular	121	IP1	145	Frontoparietal
2	ProS	121	Visual	62	9-46d	86	Cingulo-opercular	122	PFm	149	Frontoparietal
3	DVT	142	Visual	63	43	99	Cingulo-opercular	123	p10p	170	Frontoparietal
4	MST	2	Visual	64	PFcm	105	Cingulo-opercular	124	p47r	171	Frontoparietal
5	V6	3	Visual	65	PoI2	106	Cingulo-opercular	125	A1	24	Auditory
6	V2	4	Visual	66	FOP4	108	Cingulo-opercular	126	52	103	Auditory
7	V3	5	Visual	67	MI	109	Cingulo-opercular	127	RI	104	Auditory
8	V4	6	Visual	68	FOP1	113	Cingulo-opercular	128	TA2	107	Auditory
9	V8	7	Visual	69	FOP3	114	Cingulo-opercular	129	PBelt	124	Auditory
10	V3A	13	Visual	70	PFop	147	Cingulo-opercular	130	MBelt	173	Auditory
11	V7	16	Visual	71	PF	148	Cingulo-opercular	131	LBelt	174	Auditory
12	IPS1	17	Visual	72	PoI1	167	Cingulo-opercular	132	A4	175	Auditory
13	FFC	18	Visual	73	FOP5	169	Cingulo-opercular	133	7m	30	Default mode
14	V3B	19	Visual	74	PI	178	Cingulo-opercular	134	POS1	31	Default mode
15	LO1	20	Visual	75	a32pr	179	Cingulo-opercular	135	23d	32	Default mode
16	LO2	21	Visual	76	p24	180	Cingulo-opercular	136	v23ab	33	Default mode
17	PIT	22	Visual	77	PEF	11	Dorsal attention	137	d23ab	34	Default mode
18	MT	23	Visual	78	7PL	46	Dorsal attention	138	31pv	35	Default mode
19	LIPv	48	Visual	79	MIP	50	Dorsal attention	139	a24	61	Default mode
20	VIP	49	Visual	80	LIPd	95	Dorsal attention	140	d32	62	Default mode
21	PH	138	Visual	81	6a	96	Dorsal attention	141	p32	64	Default mode
22	V6A	152	Visual	82	PFt	116	Dorsal attention	142	10r	65	Default mode
23	VMV1	153	Visual	83	AIP	117	Dorsal attention	143	47m	66	Default mode
24	VMV3	154	Visual	84	PHA3	127	Dorsal attention	144	8Av	67	Default mode
25	V4t	156	Visual	85	TE2p	136	Dorsal attention	145	8Ad	68	Default mode
26	FST	157	Visual	86	PHT	137	Dorsal attention	146	9m	69	Default mode
27	V3CD	158	Visual	87	PGp	143	Dorsal attention	147	8BL	70	Default mode
28	LO3	159	Visual	88	IP0	146	Dorsal attention	148	9p	71	Default mode
29	VMV2	160	Visual	89	55b	12	Language	149	10d	72	Default mode
30	VVC	163	Visual	90	PSL	25	Language	150	47l	76	Default mode
31	4	8	Somatomotor	91	SFL	26	Language	151	9a	87	Default mode
32	3b	9	Somatomotor	92	STV	28	Language	152	10v	88	Default mode
33	5m	36	Somatomotor	93	44	74	Language	153	10pp	90	Default mode
34	5L	39	Somatomotor	94	45	75	Language	154	OFC	93	Default mode
35	24dd	40	Somatomotor	95	IFJa	79	Language	155	47s	94	Default mode
36	24dv	41	Somatomotor	96	IFSp	81	Language	156	EC	118	Default mode
37	7AL	42	Somatomotor	97	STGa	123	Language	157	PreS	119	Default mode
38	7PC	47	Somatomotor	98	A5	125	Language	158	H	120	Default mode
39	1	51	Somatomotor	99	STSda	128	Language	159	PHA1	126	Default mode
40	2	52	Somatomotor	100	STSdp	129	Language	160	STSvp	130	Default mode
41	3a	53	Somatomotor	101	TPOJ1	139	Language	161	TGd	131	Default mode
42	6d	54	Somatomotor	102	TGv	172	Language	162	TE1a	132	Default mode
43	6mp	55	Somatomotor	103	RSC	14	Frontoparietal	163	TE2a	134	Default mode
44	6v	56	Somatomotor	104	POS2	15	Frontoparietal	164	PGi	150	Default mode
45	OP4	100	Somatomotor	105	7Pm	29	Frontoparietal	165	PGs	151	Default mode
46	OP1	101	Somatomotor	106	8BM	63	Frontoparietal	166	PHA2	155	Default mode
47	OP2-3	102	Somatomotor	107	8C	73	Frontoparietal	167	31pd	161	Default mode
48	FOP2	115	Somatomotor	108	a47r	77	Frontoparietal	168	31a	162	Default mode
49	Ig	168	Somatomotor	109	IFJp	80	Frontoparietal	169	25	164	Default mode
50	FEF	10	Cingulo-opercular	110	IFSa	82	Frontoparietal	170	s32	165	Default mode
51	5mv	37	Cingulo-opercular	111	p9-46v	83	Frontoparietal	171	STSva	176	Default mode
52	23c	38	Cingulo-opercular	112	a9-46v	85	Frontoparietal	172	TE1m	177	Default mode
53	SCEF	43	Cingulo-opercular	113	a10p	89	Frontoparietal	173	PCV	27	Multimodal
54	6ma	44	Cingulo-opercular	114	11l	91	Frontoparietal	174	TPOJ2	140	Multimodal
55	7Am	45	Cingulo-opercular	115	13l	92	Frontoparietal	175	TPOJ3	141	Multimodal
56	p24pr	57	Cingulo-opercular	116	i6-8	97	Frontoparietal	176	PeEc	122	Multimodal
57	33pr	58	Cingulo-opercular	117	s6-8	98	Frontoparietal	177	TF	135	Multimodal
58	a24pr	59	Cingulo-opercular	118	AVI	111	Frontoparietal	178	Pir	110	Orbito-affective
59	p32pr	60	Cingulo-opercular	119	TE1p	133	Frontoparietal	179	AAIC	112	Orbito-affective
60	6r	78	Cingulo-opercular	120	IP2	144	Frontoparietal	180	pOFC	166	Orbito-affective

**Figure 1.**
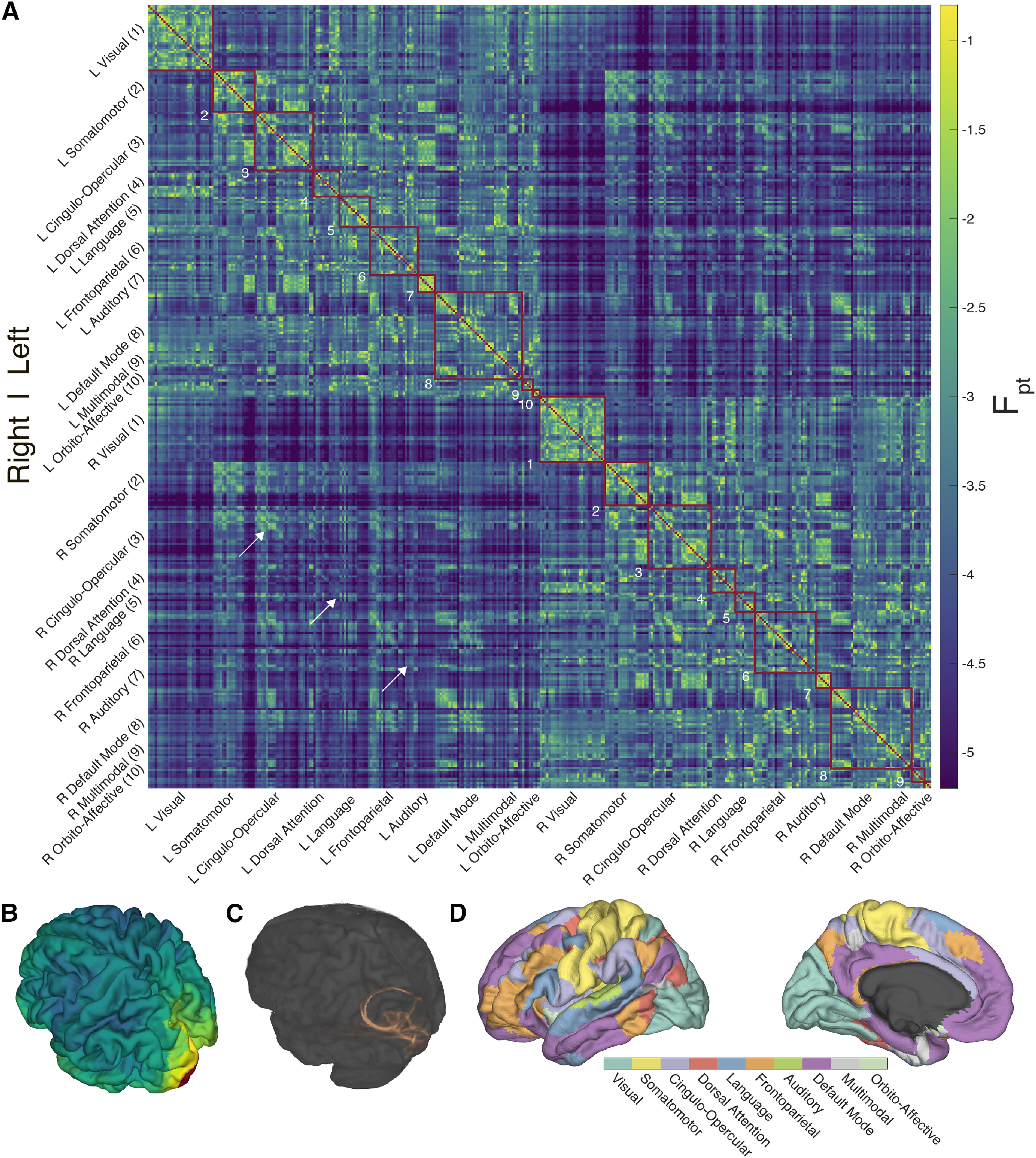
Probabilistic diffusion tractography structural connectome of the human cortex. ***A***, Group average (*N* = 1065) structural connectivity matrix consisting of the 360 HCP-MMPS1.0 atlas parcels organized into 10 functional networks. Raw streamline counts are fractionally scaled yielding the log probability F_pt_. The white arrows highlight the diagonal which contains contralateral homologs. ***B***, The first row of the connectivity matrix, showing connection probabilities from left V1 to all other parcels, projected onto the fsaverage template cortex. ***C***, Single subject (100307) volume ray casting visualization of left V1-originating streamline probabilities within the skull-stripped T1-weighted structural MR volume. ***D***, Ten functional networks, adapted from Ji et al. (2019), within HCP-MMPS1.0 atlas. These are indicated by red boxes in panel ***A***.

The Idx indices refer to the parcel order in Figure 1*A*. The Orig. indices refer to the original parcel order presented in Glasser et al. (2016). All indices refer to the left hemisphere, adding 180 yields the homologous right hemisphere indices.

### Probabilistic tractography

All analysis of diffusion imaging data were performed with FSL (Behrens et al., 2007; Jenkinson et al., 2012) release 6.0.1. Analyses were performed identically for each subject and broadly follow (Burns, 2014). The diffusion and bedpostX precursor directories made available from the HCP database were used as inputs without modification. The WU-Minn HCP diffusion data are corrected for eddy currents and movement with FSL eddy (Andersson and Sotiropoulos, 2016). Subjects’ estimated displacement over time from their initial position is written to the eddy_restricted_movement_rms output. Using these data, a scalar index of each subject’s motion was derived by integrating their displacement over time.

Fractional anisotropy (FA) analysis was performed using dtifit. The resulting FA volumes were not analyzed but only used for registering the FreeSurfer and dMRI volumes (flirt), as is necessary to map the parcel masks into dMRI space (probtrackx2 arguments –xfm –seedref). Non-invasive probabilistic tractography was performed with probtrackx2 in voxel-by-parcel mode (–os2t –s2tastext). In this configuration, the number and length of streamlines (–ompl –opd) is estimated from each voxel in the seed parcel to each target parcel as a whole. To aid parallelization of these computationally intensive processes, the list of target parcels (–targetmasks) was quartered into four sublists. Therefore, probtrackx2 was invoked 1440 times per subject, estimating the connectivity between 1 seed parcel and 90 target parcels in each invocation. The default ½ voxel step length, 5000 samples and 2000 steps were used (–steplength 0.5 -P 5000 -S 2000). To avoid artifactual loops, streamlines that loop back on themselves were discarded (-l) and tractography was constrained by a 90° threshold (-c 0) for maximal curvature between successive steps. Within-parcel connectivity and cotico-subcortical connectivity were not examined in this study. All *post hoc* analyses and visualization of connectivity data were performed in MATLAB 2019b (MathWorks) except for Figure 1*C*, which was rendered in fsleyes.

### Normalization and symmetrization

Raw streamline counts were averaged across all subjects, then normalized and symmetrized following procedure developed for non-human primate histologic tracing (Donahue et al., 2016; Theodoni et al., 2020). Briefly, fractionally scaled values are defined as the ratio of the number of streamlines originating at parcel A and terminating at parcel B to the total number of streamlines that either originate at parcel A or terminate at parcel B while excluding within-parcel connections.

(1) $$F\left(DTI_{i,j}\right)=\frac{DTI_{i,j}}{\sum_{x=1}^{N}DTI_{i,x}+\sum_{y=1}^{N}DTI_{y,j}},where\;x\neq i\text{ \& }y\neq j.$$

Fractional scaling is one of severalplausible normalization strategies. Because we used 5000 samples (-P 5000) and voxel-by-parcel mode (–os2t) in our probtrackX invocation, the maximum possible raw streamline count between any two parcels is 5000N where *N* is the number of voxels in the seed parcel. Note that because, for probtrackX, all parcels were defined as a single layer of 1-mm isotropic voxels at the white matter–gray matter interface, Ni is also equivalent to the area of the seed parcel, in mm^2^. As shown in Extended Data Figure 1-1. We examined four strategies for normalizing the raw streamline counts: (1) dividing by the number of samples, 5000; (2) dividing by the number of samples and seed area, 5000N_i_; (3) dividing by the number of samples and the areas of both the seed and target parcels, 5000N_i_^0.5^N_j_^0.5^; and (4) fractional scaling (see Eq. 1). These approaches yield similar connectivity matrices, distributions of pairwise connectivity, and rates of connectivity fall-off with fiber tract distance. The choice of normalization does shift the absolute scale of pairwise connectivity strengths, but as this effect is mostly homogenous across all connections, subsequent analyses are not greatly affected. The correlation coefficient of connectivity strengths between normalization techniques exceeds 0.97 for all pairwise comparisons, and exceeds 0.99 if the samples-only normalization approach is excluded (data not shown).

10.1523/ENEURO.0416-20.2020.f1-1 Extended Data Figure 1-1 Comparison of normalization methods. Shown are the (***A***) connectivity matrices, (***B***) distributions of pairwise connectivity, (***C***) the pre-log distribution of F_pt_ (***D***) relationships between connectivity and fiber tract length for four normalization methods. Download Figure 1-1, TIF file.

While diffusion tractography is not sensitive to the directionality of connections, because parcel A to B and parcel B to A streamlines are computed separately minor asymmetries arise. Connectivity matrix symmetry is enforced by taking the arithmetic mean of the A-B and B-A fractionally scaled connection weights.

(2) $$F_{i,j}=\frac{F_{i,j}+F_{j,i}}{2}.$$

Because probabilistic tractography values span several orders of magnitude, and are approximately log-normally distributed (Extended Data Fig. 1-1*B*), data were log-transformed (log_10_) before subsequent analyses. The CCEP and rs-fMRI connectivity matrices were (re)normalized following the same procedure. However, the rsMRI connectivity values were not log-transformed, because these data are already approximately normally distributed, if bimodal, in linear space).

### Network theory metrics

All network theoretic measures were computed in MATLAB using the Brain Connectivity Toolbox, 2019-03-03 release (Rubinov and Sporns, 2010). It is available at http://www.brain-connectivity-toolbox.net or https://www.nitrc.org/projects/bct. The definitions for the metrics used (for binary and undirected networks) are repeated below.

#### Precursor measures

(3) $$d_{i,j}=\underset{a_{u,v}\in g_{i↔j}}{\sum }a_{u,v},$$

where *d_i,j_* is the shortest path length, a basis for measuring integration, between nodes *i* and *j*, *N* is the set of all nodes in the network, *n* is the number of nodes, and *a_u,v_* is the binarized connectivity between nodes *u* and *v*.

(4) $$t_{i}=\frac{1}{2}\underset{j,h\in N}{\sum }a_{i,j}a_{i,h}a_{j,h},$$

where *t_i_* is the number of triangles, a basis for measuring integration, around node *i*.

(5) $$k_{i}=\underset{j\in \text{N}}{\sum }a_{i,j},$$

where *k_i_* is the number of degrees, or number of links, connected to node *i*.

#### Mean clustering coefficient (MCC)

(6) $$C_{i}=\frac{1}{n}\underset{i\in \text{N}}{\sum }\frac{2t_{i}}{k_{i}(k_{i}-1)\prime },$$

where *C_i_* is the clustering coefficient of node *i*. (*C_i_* = 0 for *k_i_* < 2; Watts and Strogatz, 1998).

(7) $$\text{MCC}=\frac{1}{n}\underset{i\in \text{N}}{\sum }C_{i}.$$

#### Characteristic path length (CPL)

(8) $$L_{i}=\frac{1}{n}\underset{i\in \text{N}}{\sum }\frac{\sum_{j=1,j\neq i}^{n}d_{i,j}}{n-1},$$

where *L_i_* is the number of the average distance between node *i* and all other nodes (Watts and Strogatz, 1998).

(9) $$CPL=\frac{1}{n}\underset{i\in \text{N}}{\sum }L_{i}.$$

#### Global efficiency

(10) $$E_{i}=\frac{1}{n}\underset{i\in \text{N}}{\sum }\frac{\sum_{j\in \text{N},j\neq i}^{n}d_{i,j}^{-1}}{n-1},$$

where *E_i_* is the efficiency of node *i*.

(11) $$E=\frac{1}{n}\underset{i\in \text{N}}{\sum }L_{i},$$

where *E* is the global efficiency of the network (Latora and Marchiori, 2001).

#### Modularity

(12) $$Q=\frac{1}{l}\underset{i,k\in \text{N}}{\sum }\left(a_{i,j}-\frac{k_{i}k_{j}}{l}\right)\delta_{m_{i},m_{j}},$$

Where *l* is the number of links in the network, *m_i_* is module containing node *i*, *δ_mi,mj_* = 1 if *m_i_* = *m_j_*, and 0 otherwise, and *Q* is the global efficiency of the network (Newman, 2004).

#### γ (normalized MCC)

(13) $$\gamma =\frac{MCC}{MCC_{rand}},$$

where *MCC_rand_* is the *MCC* of a random network of the same statistical makeup.

#### λ (normalized CPL)

(14) $$\lambda =\frac{CPL}{CPL_{rand}},$$

where *CPL_rand_* is the *CPL* of a random network of the same statistical makeup. Note that this measure is unrelated to the length constant λ.

#### Small-worldness

(15) $$S=\frac{\gamma }{\lambda },$$

where *S* is the network small-worldness (Humphries and Gurney, 2008).

#### Transitivity

(16) $$T=\frac{\sum_{i\in \text{N}}2t_{i}}{\sum_{i\in \text{N}}k_{i}\left(k_{i}-1\right)},$$

where *T* is the transitivity of the network (Newman, 2003).

#### Assortativity

(17) $$r=\frac{l^{-1}\sum_{(i,j)\in \text{L}}k_{i}k_{j}-{\left[l^{-1}\sum_{(i,j)\in \text{L}}\frac{1}{2}\left(k_{i}+k_{j}\right)\right]}^{2}}{l^{-1}\sum_{(i,j)\in \text{L}}\frac{1}{2}\left(k_{i}^{2}+k_{j}^{2}\right)-{\left[l^{-1}\sum_{(i,j)\in \text{L}}\frac{1}{2}\left(k_{i}+k_{j}\right)\right]}^{2}},$$

where *L* is the set of all links and *r* is the assortativity coefficient of the network (Newman, 2003).

#### Network density

(18) $$D=\frac{l}{n^{2}-n},$$

where *D* is the density of the network before thresholding and binarization.

### Data availability

Individual and group average connectivity matrices as well as all other figure source data can be found at https://doi.org/10.5281/zenodo.4060485 (https://zenodo.org/record/4060485). These data also include statistical uncertainty (95% confidence intervals) for results not listed in Table 3. The preprocessed HCP data using in this study was retrieved from https://db.humanconnectome.org and the preprocessing code used to create these files is available at https://github.com/Washington-University/HCPpipelines. The source code for FSL, including probtrackx2 is available from https://fsl.fmrib.ox.ac.uk/fsl/fslwiki/FSL. Network theory measures were computed with the brain connectivity MATLAB toolbox whose source code is available from http://www.brain-connectivity-toolbox.net.

**Table 3:** Statistics and uncertainty

Location	Data structure	Test or analysis	*N*	Uncertainty [CI_95%_]
Extended Data Fig. 1-1*D*	Gaussian predictor Exponential response	Nonlinear regression (iterative optimization)	64,620 64,620 64,620 64,620	λ = 23.8 [23.5, 24.0] λ = 22.8 [22.7, 22.9] λ = 22.2 [22.1, 22.2] λ = 23.4 [23.3, 23.6]
Fig. 2*A*	Gaussian predictor Exponential response	Nonlinear regression (iterative optimization)	16,110	λ = 23.1 [22.8, 23.3]
Fig. 2*B*	Gaussian predictor Exponential response	Nonlinear regression (iterative optimization)	16,110	λ = 23.9 [23.7, 24.2]
Fig. 2*C*	Gaussian predictor Exponential response	Nonlinear regression (iterative optimization)	32,400	λ = 32.8 [32.5, 33.0]
Fig. 2*D*	Gaussian predictor Exponential response	Nonlinear regression (iterative optimization)	64,620	λ = 23.4 [23.3, 23.6]
Extended Data Fig. 2-2*B*	Gaussian predictor Gaussian response	Linear correlation	1065	*r* = −0.14 [−0.20, −0.08]
Fig. 3*D*	Gaussian predictor Exponential response	Nonlinear regression (iterative optimization)	12,924	λ = 27.8 [27.4, 28.2]
Fig. 3*F*	Gaussian predictor Gaussian response	Linear correlation	1065	*r* = 0.70 [0.67, 0.73]
Fig. 4*C*	Gaussian predictor Gaussian response	Linear correlation	80	*r* = 0.35 [0.14, 0.53]
Fig. 8*A*	Gaussian predictor Gaussian response	Linear correlation	16,110 16,110 32,400	*r* = −0.10 [−0.12, −0.09] *r* = −0.12 [−0.13, −0.10] *r* = −0.11 [−0.12, −0.10]
Fig. 8*B*	Gaussian predictor Gaussian response	Linear correlation	351 351 66 91 231 231 28 780 780 10	*r* = −0.17 [−0.27, −0.06] *r* = −0.13 [−0.23, −0.02] *r* = −0.41 [−0.60, −0.19] *r* = −0.26 [−0.44, −0.06] *r* = −0.30 [−0.42, −0.18] *r* = −0.30 [−0.40, −0.17] *r* = −0.56 [−0.77, −0.24] *r* = −0.12 [−0.19, −0.05] *r* = −0.17 [−0.24, −0.10] *r* = −0.74 [−0.93, −0.20]
Fig. 9*B*	Gaussian predictor Gaussian response	Linear correlation	19,667 19,667 64,620	*r* = 0.43 [0.42, 0.44] *r* = 0.23 [0.21, 0.24] *r* = 0.06 [0.05, 0.07]
Extended Data Fig. 9-1	Gaussian predictor Gaussian response	Linear correlation	8483 8483 16,110 8370 8370 16,110	*r* = 0.42 [0.40, 0.44] *r* = 0.22 [0.20, 0.24] *r* = 0.06 [0.05, 0.07] *r* = 0.40 [0.38, 0.42] *r* = 0.22 [0.20, 0.24] *r* = 0.11 [0.10, 0.13]

Where multiple uncertainties are listed for a figure panel, they correspond to the statistics read left-to-right, top-to-bottom in that panel. For Figure 8*B*, only uncertainties for significant correlations are listed. Uncertainties for Figures 6-8, 10 are not shown. Extended Data Figure 6-1 contains bootstrapped 95% confidence intervals for the 180 means shown in Figure 6, *n* = 179. Figure 7 shows bootstrapped 95% confidence intervals in gray; the values of these intervals for all distance bins are available in the figure source data at https://doi.org/10.5281/zenodo.4060485. For Figure 10, means across shuffled matrices are only necessary to account for arbitrary ordering among tied edge weights, and the bootstrapped 95% confidence intervals for these means are vanishingly small. The values of these intervals at all network densities are also included in the figure source data. For nonlinear regressions, confidence intervals are estimated using *R*^−1^, the inverse *R* factor from *QR* decomposition of the Jacobian, the degrees of freedom for error, and the root mean squared error. For linear correlations, the confidence intervals are based on an asymptotic normal distribution of 0.5*log((1+r)/(1–r)), with an approximate variance equal to 1/(*N* – 3). For descriptive statistics, e.g., means, empirical 95% confidence intervals are estimated by bootstrapping with 2000 iterations.

## Results

### A whole-cortex structural connectome

Figure 1*A* shows the group average parcel to parcel and probabilistic diffusion tractography connectome. This matrix consists of connectivity among the 360 cortical parcels of the HCP-MMP1.0 atlas. Using left V1 connectivity as an example, Figure 1*B* illustrates the spatial mapping of the connectivity matrix to the cortex, and Figure 1*C* shows a rendering of streamline paths for one subject. The cortical parcels are further organized into 10 functional groups per hemisphere modified from Ji et al. (2019). These larger functional groupings are shown in Figure 1*D*. The raw probabilistic tractography streamline counts have been normalized by fractionally scaling (Eq. 1) into log probabilities (F_pt_) following procedures developed for tracing non-human primate connectivity. As dMRI reveals structural connections, the network is undirected and therefore symmetric. The main diagonal is masked as intraparcel connectivity was not examined in this study. The upper left quadrant shows connectivity among the 180 parcels of the left hemisphere, the lower right quadrant the connectivity within the right hemisphere. The upper right and lower left quadrants are duplicates and show the interhemispheric, or callosal, connections. The 180^th^ (or half-) diagonal is clearly visible (white arrows); this shows the connectivity between homologous parcels in the right and left hemispheres, which is greater than non-homologous callosal connectivity for most parcels.

After log_10_ transformation, F_pt_ connectivity among all parcel pairs is approximately Gaussian in distribution with mean 3.903 with 95% confidence interval (CI_95%_) of [3.897, 3.910], standard deviation 0.8111 (CI_95%_ = [0.806, 0.816]), skewness 0.627 (CI_95%_ = [0.608, 0.644]), and kurtosis 3.605 (CI_95%_ = [3.560, 3.650]). In addition to bringing the range of F_pt_ values into the same order of magnitude, log_10_ transformation is justified as it brings the distribution’s skewness significantly closer to zero (pre-log_10_: 9.047, CI_95%_ = [8.719, 9.469]), and kurtosis significantly closer to three, pre-log_10_: 103.684 (CI_95%_ = [93.991, 117.026]) thus bringing the distribution closer to normality. See Extended Data Figure 1-1*B*,*C* for a graphical comparison. Empirical confidence intervals were estimated via bootstrapping with 2000 iterations. The values of the group average and individual probabilistic dMRI connectivity matrices, as well as all other figure source data can be found at https://doi.org/10.5281/zenodo.060485.

### Tract length strongly predicts connectivity strength, with exponential decay

**Figure 2.**
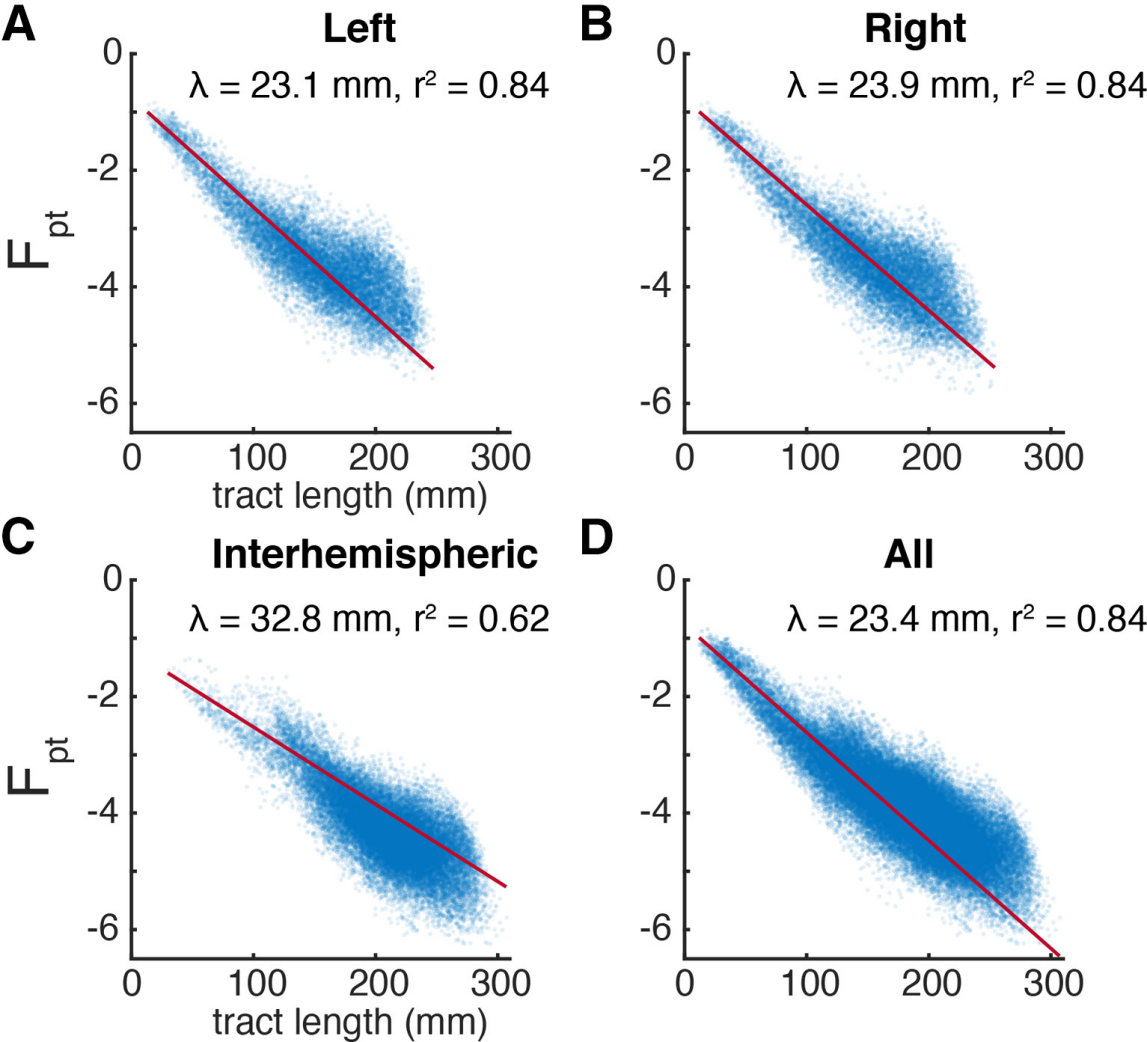
Connectivity strength exponential decays with fiber tract length. ***A***, ***B***, Connections within the right and left hemispheres, respectively. ***C***, Connections between the right and left hemisphere. ***D***, All connections. Each marker represents a pair of parcels. Red traces show the least-squares exponential fit; inset are the length constant λ and *r*^2^ of this fit. Note that F_pt_ is log-transformed making these axes effectively semi-log.

In addition to the connection strength, diffusion tractography estimates the fiber tract length between all pairs of parcels. As shown in Figure 2, structural connectivity (10^F_pt_) falls off as an exponential function of fiber tract length with the form 10^F_pt_ = α*e^-d/λ^ where λ is the length constant, α the scaling coeffect, and d the tract length. Alternative functional forms were examined (Extended Data Fig. 2-1), but the exponential was selected for parsimony, goodness-of-fit, and concordance with histologic tracing data (see Discussion). Note that λ is sometimes reported in inverted units of mm^−1^ (Markov et al., 2013; Theodoni et al., 2020), but we here use the λ convention from neuronal cable theory (Dayan and Abbott, 2001), which has more intuitive units (mm); the conventions are conceptually equivalent. For the group-average connectome, λ = 23.4 mm and the least-squares exponential fit explains 84% of the variance in 10^F_pt_ across all parcel pairs. Callosal connectivity, when isolated, decays more slowly with respect to tract length, λ = 32.8, and hews to the exponential expectation less consistently *r*^2^ = 0.62. Because the tracing of long fiber tracts may be hampered by poor scan quality, we investigated the effects of subjects’ motion on λ. For each subject, λ was calculated for non-zero connections in the same manner as the group average. While subjects’ motion within the scanner does reduce λ, this effect is modest, only explaining 1.96% of the intersubject variance (Extended Data Fig. 2-2).

10.1523/ENEURO.0416-20.2020.f2-1 Extended Data Figure 2-1 Alternative models for fitting connectivity strength as a function of fiber tract length. Each gray marker shows the average pair-wise Fpt between two parcels and fiber tract length between them, as also shown in Figure 2*D*. The colored traces show maximum likelihood estimates for several listed functional forms. The AIC, AICc, and aBIC columns contain the Akaike, corrected Akaike, and Bayesian information criteria, respectively. While the Gaussian fits explain slightly more variance and have a slightly lower AIC than the exponential fit, the exponential has fewer parameters and is consistent with histological non-human primate evidence (Markov et al., 2013; Donahue et al., 2016; Theodoni et al., 2020). Download Figure 2-1, TIF file.

10.1523/ENEURO.0416-20.2020.f2-2 Extended Data Figure 2-2 Effect of motion during the dMRI scan. ***A***, Time course of displacement relative to initial position for one subject (996782). The six runs of the HCP dMRI protocol can be seen. ***B***, Exponential fall-off coefficient λ is only modestly affected by motion, *r* = 0.140, *p* = 4.6E-6. Each marker represents a subject. Download Figure 2-2, TIF file.

### Interindividual variability

**Figure 3.**
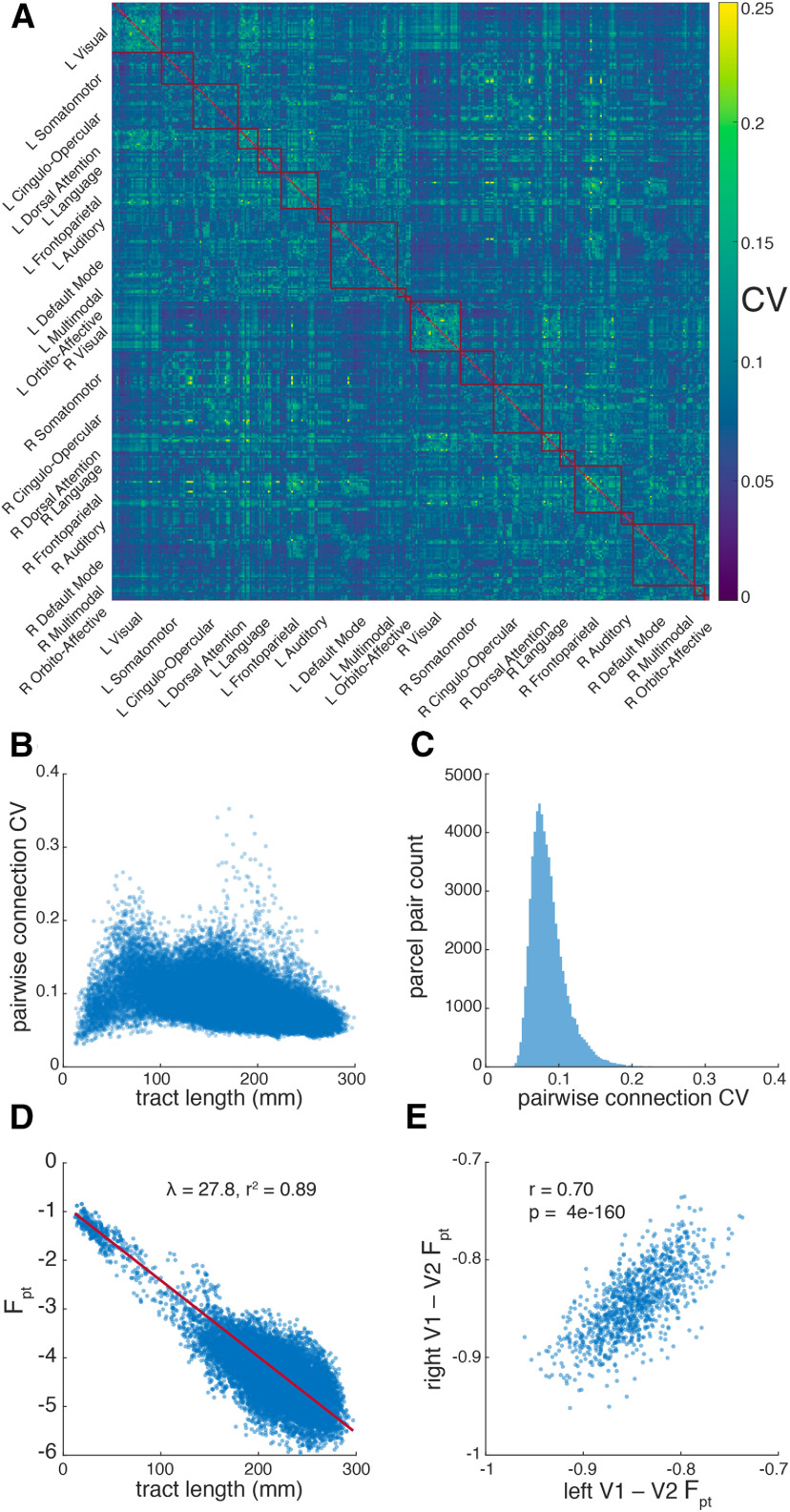
Interindividual variability. Shown are (***A***) the matrix of connectivity coefficients of variation (CV) across subjects (***B***) pairwise CV versus fiber tract length, (***C***) the distribution of CV across all connections, (***D***) the F_pt_ versus fiber tract length for the connections in the highest quintile of interindividual consistency, and (***E***) the F_pt_ of right hemisphere V1–V2 connection in all subjects versus left hemisphere V1–V2 connection. In panels ***B***, ***D*,** each marker represents a sample statistic for a connection between two parcels. ***E***, Each marker represents an individual subject. ***D***, The red trace show the least-squares exponential fit, and inset is the length constant λ and *r*^2^ of this fit. Note that F_pt_ is log-transformed making this panel’s axes effectively semi-log. In panel ***E***, the *r*^2^ of the least-squares linear fit is reported.

The interindividual variability of connectivity was assessed by deriving the across-subject coefficient of variation (CV) for each pairwise connection F_pt_ (Fig. 3). For this analysis, the normalization, symmetrization, and log_10_-transformation of raw connectivity values were performed on each subject. Pairwise connections with zero streamlines were not log-transformed to avoid infinities. While there is no clear relationship between fiber tract distance and interindividual variability, the most consistent connection appears in two clusters of around 50–100 and 170–225 mm (Fig. 3*B*). When the most consistent quintile of connections is isolated (Roberts et al., 2017), connectivity falls off more slowly with tract distance, with λ increasing to ∼28 mm (Fig. 3*D*). Since the proportional size of V1/V2 varies ∼3-fold across individuals and is highly heritable (Yoon et al., 2019), we hypothesized that the ipsilateral V1–V2 connection would also be highly variable, with that variability being correlated across hemispheres. Indeed, we find that the ipsilateral V1–V2 connection is very strong, with ∼1.8-fold variability which is strongly correlated across hemispheres (*r* = 0.70). The scatter-plot of right versus left F_pt_ values for this connection across subjects (Fig. 3*F*) does not reveal obvious outliers which would be indicative of subject-specific artifacts. This analysis of interindividual variability should be considered preliminary. The WU-Minn HCP dataset is rich in individual data, including the NIH neuropsychological toolbox (Gershon et al., 2013), twin and non-twin siblings subsets, and genotypic data (dbGaP phs001364.v1.p1), although the latter two data types are only available by application to ensure subject anonymity. With access to these data, a full examination of interindividual variability, including assessing the heritability and genetic correlates of the strength of specific connections could be made.

### Probabilistic dMRI tract tracing in humans reasonably corresponds with histologic fiber tracing in macaques

**Figure 4.**
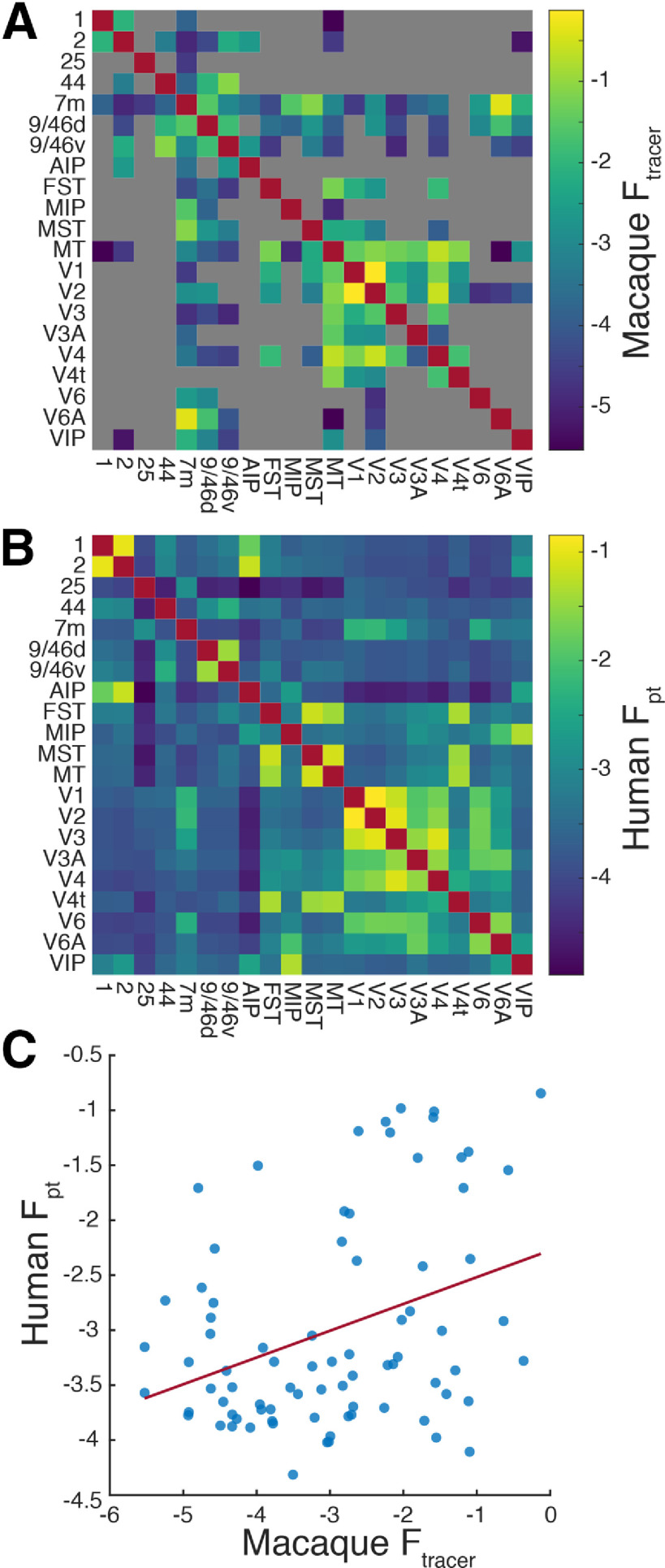
Comparison of human diffusion tractography and macaque retrograde tracing connectomes. Subset of homologous parcels in the human HCP-MMPS1.0 and macaque fv91 atlas. ***A***, Macaque group-average retrograde tracer derived structural connectome, gray indicates missing data. ***B***, Human probabilistic diffusion tractography connectome. ***C***, Pairwise correlation between macaque and human structural connectivity, *r* = 0.35, *p* = 0.0013.

The development of both the HCP-MMP1.0 human cortical atlas (Glasser et al., 2016) and FV91 macaque parcellation scheme (Felleman and Van Essen, 1991) were led by David Van Essen and the parcel definitions of the human atlas were informed by human-macaque homology. As such, the parcel names of these atlases have considerable overlap, particularly for visual and visual association areas as well as the non-visual parcels 1, 2, 25, and 44. We therefore assumed that parcels with the same name were roughly homologous and limited the scope of the interspecies comparison to these parcels. Furthermore, the macaque F_Lne_ values found in Markov et al. (2014) are directly comparable to fractionally scaled F_pt_ values (Donahue et al., 2016). Comparing the pairwise connectivity between species, we found a Pearson correlation of *r *=* *0.35 (*p* = 0.0013; Fig. 4). Considering that for macaques, Donahue et al. (2016) found a within-species, between-technique correlation of *r *=* *0.59 when comparing retrograde tracing and probabilistic diffusion tractography, we find the magnitude of between-species correlation to be reasonable supporting evidence for the efficacy of the technique.

### Contralateral connectivity exceeds ipsilateral connectivity in some regions

**Figure 5.**
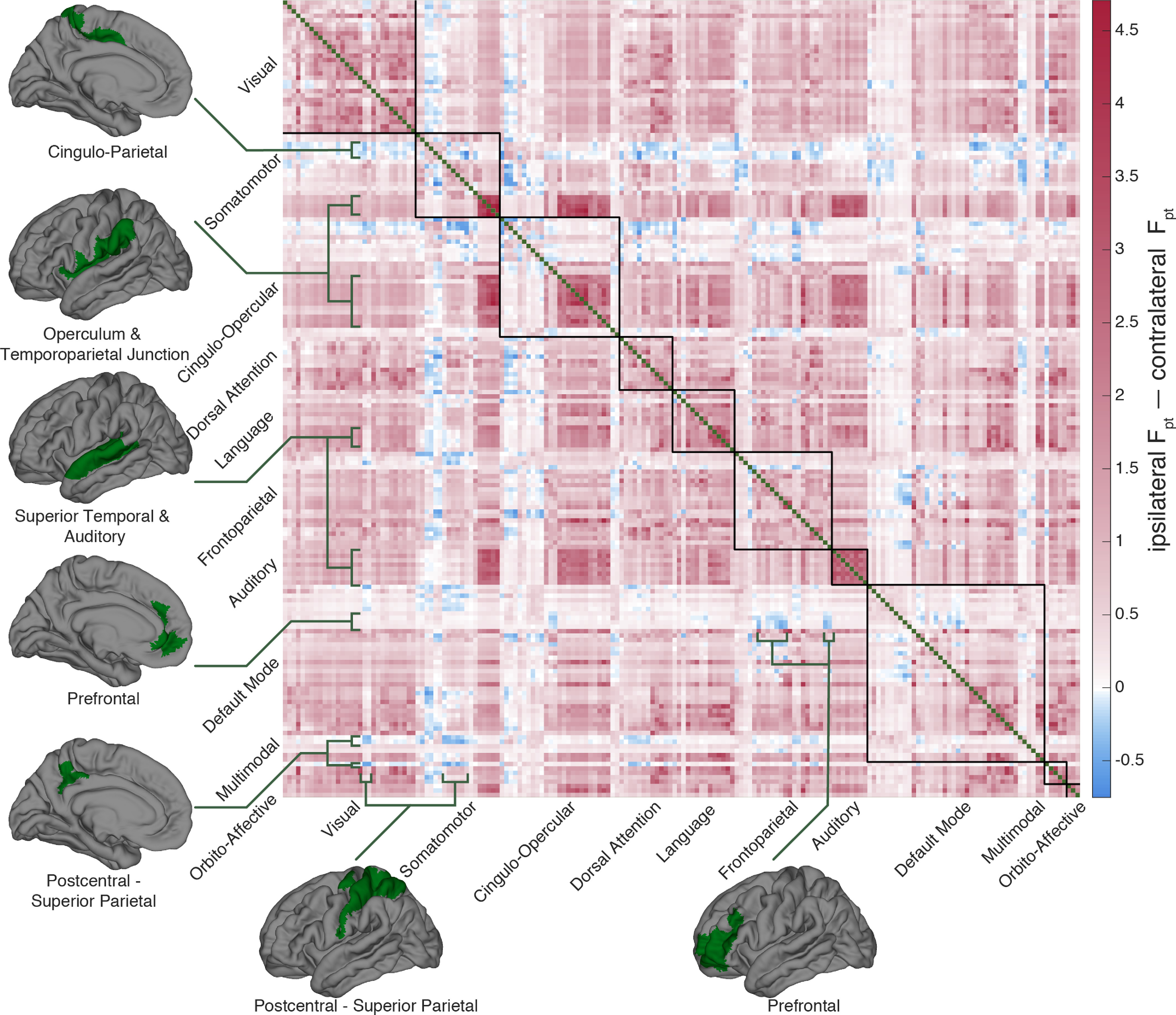
Interhemispheric connectivity. Differential connectivity between ipsilateral and contralateral connectivity. Greater ipsilateral connectivity dominates and is indicated in red. Parcel-pairs with greater contralateral connectivity than ipsilateral are blue. The green cortical patches show anatomic extent of parcel groups of notable contrast.

On the whole, cortical connectivity is dominated by ipsilateral connections. This effect is readily-observed by comparing the ipsilateral and contralateral quadrants of Figure 1*A*. However, there are exceptions to this rule. The differential connectome of ipsilateral versus contralateral connections is shown in Figure 5. This is achieved by subtracting the mean of left-right and right-left contralateral connectivity from the mean of the right and left ipsilateral connectivity, i.e., subtracting the mean of the first and third quadrants from the mean of the second and fourth. A cingulo-parietal somatomotor region (parcels 5m, 5L, 24dd, and 24dv) are more strongly connected to most contralateral cortex than ipsilateral cortex. Lateromedial connectivity in select prefrontal (a10p, a9-46v, a10p, p10p, p47r, p9-46v, 11l, IFSa, IFJp, a24, d32, p32, 10r) and postcentral-superior parietal lobule (LIPv, VIP, 7AL, 7PC, 1, 2, 3a, 6d, 31a, 31pd, PCV) regions is stronger between hemispheres than within them. We speculate that a possible commonality between these three regions is that they have been broadly implicated in the unitary processes of somatosensory object recognition, emotion, and spatial cognition, respectively. Conversely, the entire auditory network and superior temporal cortices (STGa, STSda, DTDdp, A5, and TPOJ1) as well as the operculum and temporoparietal junction (Ig, MI, FOP1-FOP5, OP1-OP4, PF, PFcm, PFop, PI, PoI1, PoI2, and 43) have pronounced hyperipsilateral connectivity, consistent with the low transmission latency required for auditory processing, the left-lateralization of language, and the right lateralization of attention.

### With the exception of some language areas, most parcels are disproportionately connected to their contralateral homologs

**Figure 6.**
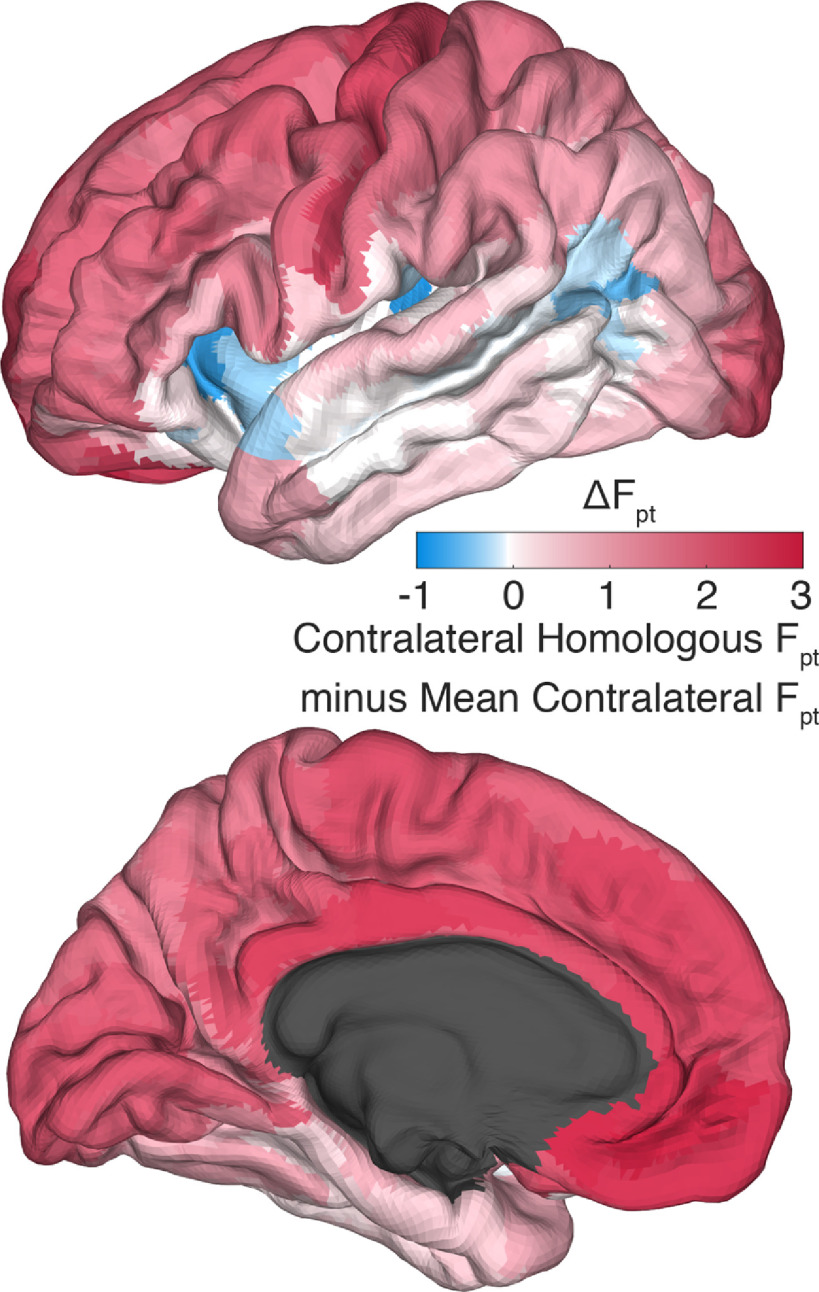
Contralateral homologs. Differential connectivity between contralateral homologous parcels versus the mean of all other contralateral parcels. Red indicates contralateral homologous connectivity greater than mean contralateral connectivity. Note that many language-implicated regions have relatively weak connectivity with their contralateral homologs.

The two hemispheres of the cortex have a high degree of functional and anatomic symmetry. It follows then that most regions will have greater connectivity to their contralateral homologs than other contralateral areas, to coordinate their overlapping processing tasks. This is hinted at by the visibility of the 180th (or half-)diagonal in Figure 1*A*. To further quantify this effect, for all 180 parcels we compared the connectivity between interhemispheric homologs to the mean of all other callosal connectivity. Bonferroni-corrected, empirical 95% confidence intervals were estimated via bootstrapping with 2000 iterations. As detailed in Extended Data Figure 6-1 and visualized in Figure 6, 147 parcels are hyperconnected to their contralateral homologs, 18 are hypoconnected, and 15 have homologous callosal connectivity not significantly different from their callosal mean connectivity. Interestingly, parcels that are not hyperconnected to their contralateral homologs are concentrated within and adjacent to the language network, consistent with the greater degree of lateralization in these areas.

10.1523/ENEURO.0416-20.2020.f6-1 Extended Data Figure 6-1 Differential connectivity between contralateral homologous parcels versus the mean of all other contralateral parcels. Confidence intervals are Bonferroni-corrected for multiple comparisons. Download Figure 6-1, DOCX file.

### The language network is hyperconnected at long distances and left lateralized

**Figure 7.**
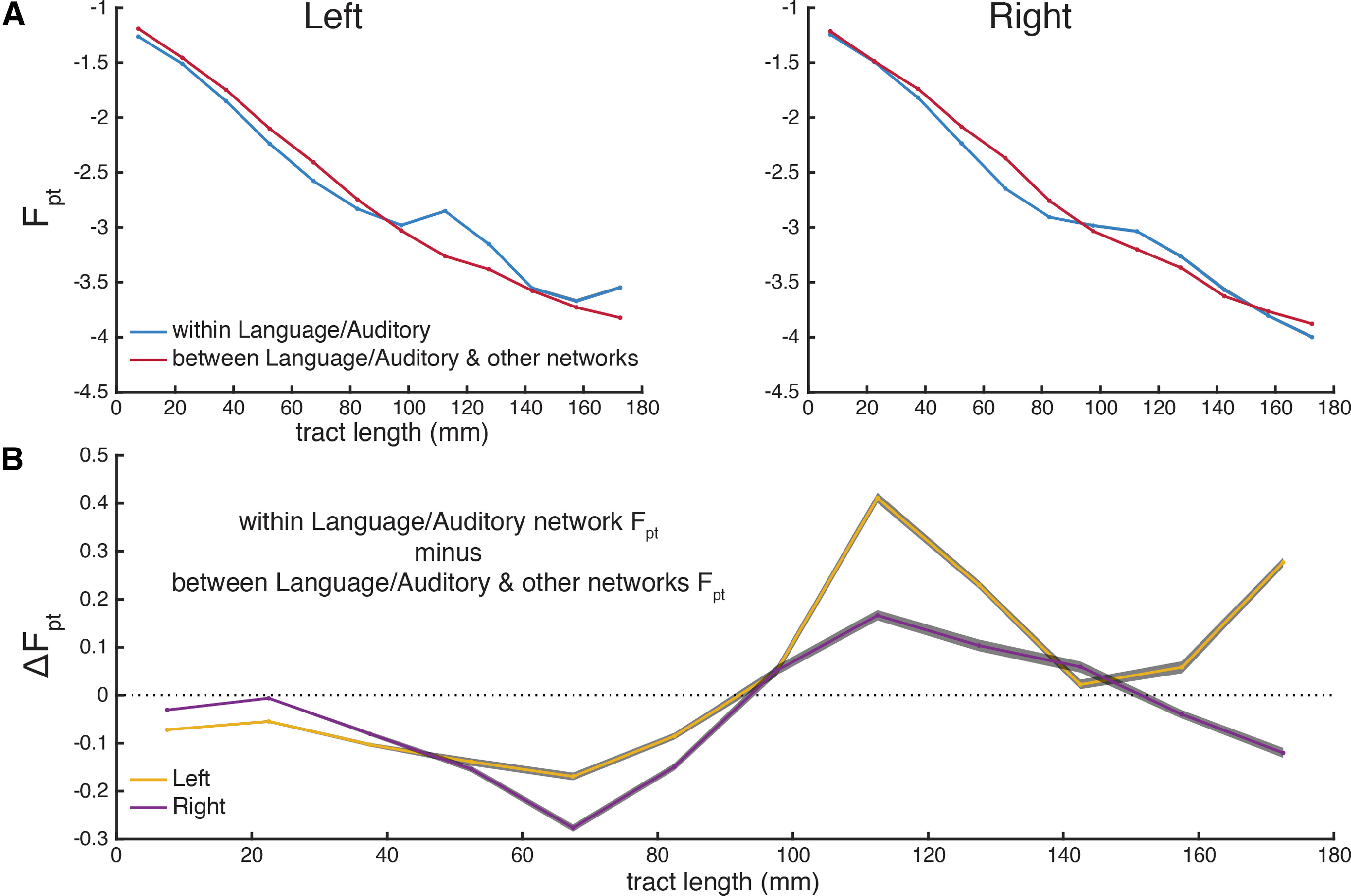
Language/auditory network hyperconnectivity and left-lateralization. ***A***, Distance-binned connectivity within the language and auditory networks compared with connectivity between the language and auditory networks and other networks, separately for the left and right hemispheres. ***B***, Differential trace for the within-connectivity and between-connectivity in both hemispheres. In both panels, gray patches show Bonferroni-corrected bootstrapped 95% confidence intervals across subjects.

In order to investigate distance-resolved left laterality in connections among language-implicated cortex, pairwise connections were binned by fiber tract length in 15-mm increments. Within each bin, connections were grouped as being within the combined language and auditory network, or between the combined networks and the rest of the cortex. For each subject, the F_pt_ of grouped connections within each bin was averaged before being log-transformed. The grand-averages of these within-language and between-language/auditory cortex in each distance bin for each hemisphere are shown in Figure 7*A*. Bonferroni-corrected, empirical 95% confidence intervals for these grand-averages were estimated via bootstrapping with 2000 iterations. Within-language connectivity is slightly attenuated at distances <100 mm, but strongly amplified at distances above 100 mm, especially ∼100– to 140-mm connections in the left hemisphere. A plurality of these are between frontal and temporoparietal language areas (18/45 connections between 100 and 140 mm). The differential traces of between-language versus within-language connectivity (Fig. 7*B*) clearly show the left-hemisphere dominance of this effect.

### Connectivity is influenced by the cortical hierarchy

Hierarchy is a central organizing principle of the cortex (Felleman and Van Essen, 1991; Markov et al., 2014; Burt et al., 2018; Theodoni et al., 2020). Higher order areas, e.g., supporting abstract processing, have low myelination, and lower order areas, e.g., supporting unimodal sensory processing, have high myelination. Furthermore, areal myelination is indexed by the ratio between T1-wieghted and T2-wieghted MRI contrast (Glasser and Van Essen, 2011). The WU-Minn HCP 1200 release includes smoothed group-average myelination indices for all vertices in the 32k grayordinate template brain. These values were averaged for each parcel in the HCP-MMP1.0 atlas (Glasser et al., 2016) to yield a group-average parcel-wise index of myelination.

**Figure 8.**
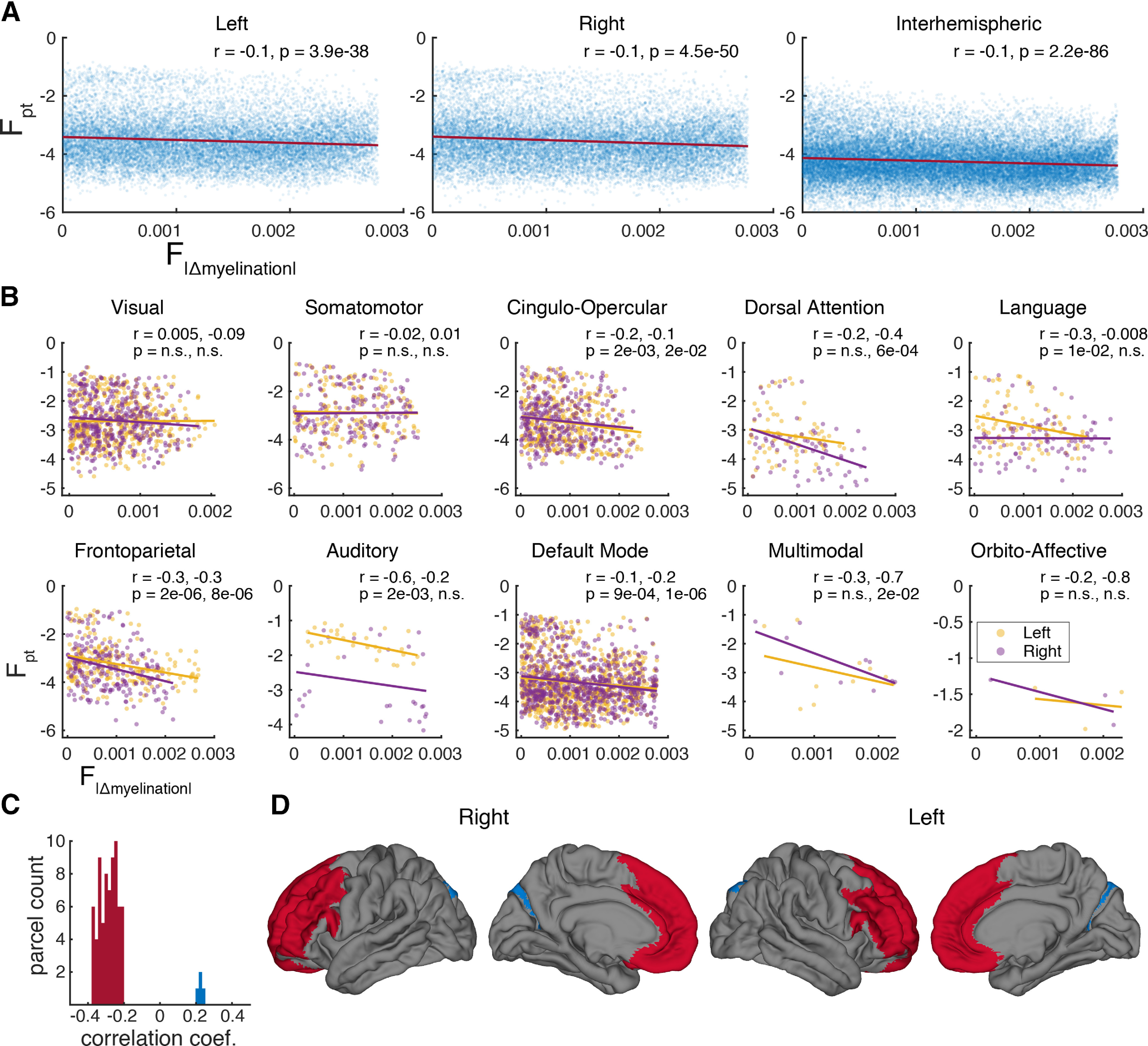
Connectivity is influenced by the cortical hierarchy. ***A***, ***B***, Connectivity is strongly predicted by hierarchical similarity in some networks and modestly predicted overall. ***A***, All connectivity versus myelination difference, including within-network and across-network connections, for the left, right, and callosal connections. For both panels, each marker represents a parcel pair. ***B***, Within-network connectivity versus myelination difference for 10 functional networks. Linear fits and correlation coefficients computed independently for the left and right hemisphere. A negative correlation indicates that parcels at similar hierarchical levels tend to be more connected. ***C***, ***D***, Higher order prefrontal areas are better connected. ***C***, Histogram of correlation coefficients between areal myelination and F_pt_ connectivity to each parcel. Only significant coefficients after Bonferroni correction are shown. Most coefficients are negative indicating high connectivity to low-myelination (i.e., higher-order) areas. ***D***, Significant negative coefficients (red) map onto bilateral prefrontal cortex. Only the bilateral DVT and V6A are show positive significant correlations (blue).

The relationship between cortical hierarchy and connectivity was assessed in two ways. We first examined whether regions of similar level in the cortical hierarchy are better connected, as predicted by Barbas (2015). An index of hierarchical similarity, F_|Δ myelination|_, was obtained for each pair of parcels by computing the pairwise difference in myelination between parcels and fractionally scaling it in the same manner as F_pt_, with smaller values indicating hierarchical closeness. The similarity matrix created by this derivation is shown in Extended Data Figure 8-1. Correlations were obtained for the left and right hemisphere as a whole as well as the colossal connections (Fig. 8*A*). In addition, for each of the twenty functional networks (10 per hemisphere) the Pearson correlation between the F_|Δ myelination|_ and F_pt_ for pairwise within-network connections was computed (Fig. 8*B*). With the exception of the interhemispheric connections, calculations were performed on the hemispheres separately to avoid the collinearity introduced by hemispheric homology.

10.1523/ENEURO.0416-20.2020.f8-1 Extended Data Figure 8-1 Myelination difference connectivity matrix. This provides an estimate for the difference in hierarchical level between cortical parcels. Values have been fractionally scaled. Note that the color scale has been reversed when compared to Figure 1, as |Δmyelination| is inversely proportional to connectivity. Download Figure 8-1, TIF file.

With the exceptions of the bilateral visual and somatomotor networks and right language network, for which there is convincingly no relationship, the preponderance of coefficients is negative, indicating that, on average, areas at similar levels of the cortical hierarchy are better connected. However, quantified in this way, the influence of hierarchy is modest, explaining ∼1% of the variance in F_pt_ overall, although perhaps 10–30% in certain subsets of parcels, such as the left auditory and language networks. The left lateralization of the influence of hierarchy in these networks is striking, as is the right-lateralization of the dorsal attention network.

Second, we investigated whether a cortical region’s hierarchical level affected its overall connectivity. For each parcel, the Pearson correlation between the parcel’s F_pt_ to all other parcels and the parcel-wise index of myelination was computed. In other words, correlation between each row of the connectome matrix and the vector of myelination indices was obtained. After Bonferroni correction for multiple comparisons, 74 of 360 parcels (Extended Data Figs. 8-2, 8-3) have connectivity significantly correlated to their myelination index and of these the vast majority (70) are negatively correlated, indicating that low myelination predicts high connectivity (Fig. 8*C*). These areas form a contiguous bilateral prefrontal network as shown in Figure 8*D*, indicating that prefrontal areas are more connected with higher cortical regions. The rare positively correlated exceptions are the left and right DVT and V6A.

10.1523/ENEURO.0416-20.2020.f8-2 Extended Data Figure 8-2 Pearson correlations between the F_pt_ from each left hemisphere parcel to all others and the target parcels’ myelination indices; *p* values are Bonferroni-corrected for multiple comparisons. Download Figure 8-2, DOCX file.

10.1523/ENEURO.0416-20.2020.f8-3 Extended Data Figure 8-3 Pearson correlations between the F_pt_ from each right hemisphere parcel to all others and the target parcels’ myelination indices; *p* values are Bonferroni-corrected for multiple comparisons. Download Figure 8-3, DOCX file.

### Probabilistic dMRI connectivity more closely resembles CCEPs than rs-fMRI

**Figure 9.**
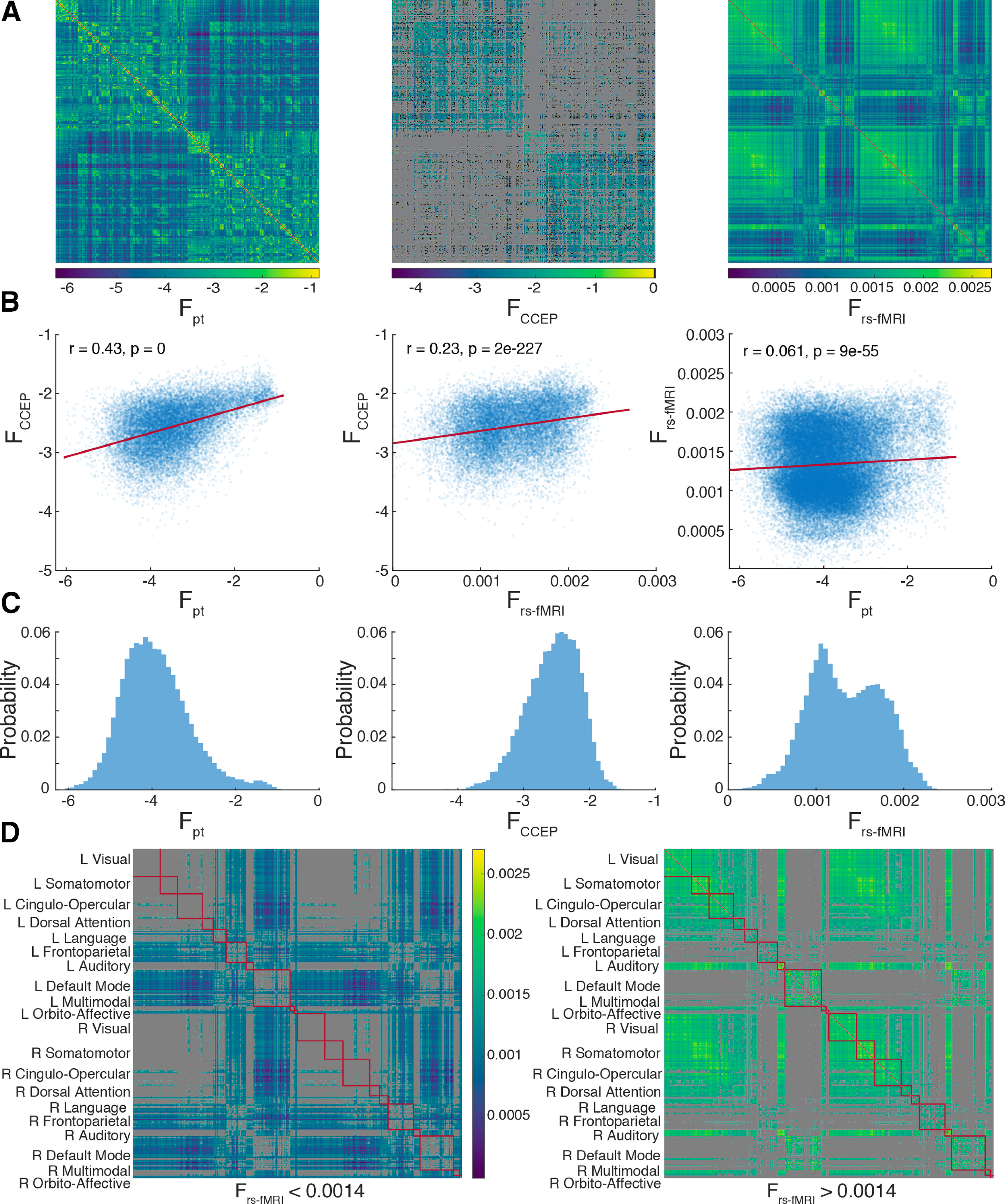
Probabilistic dMRI more closely resembles CCEPs than rs-fMRI. ***A***, Connectivity matrices for probabilistic dMRI tractography, CCEP, and rs-fMRI. For CCEPs missing data has been colored gray and pre-log zero-strength connections black. ***B***, Correlations among the three modalities. The least-squares linear fit is shown in red. ***C***, Non-zero pairwise connection strength distributions. Note that rs-fMRI connectivity values, which are not log-transformed, display two modes, separated at 0.0014. ***D***, Cortical parcels displaying lower (left) and higher (right) modes of rs-fMRI connectivity.

In order to further contextualize the dMRI connectome, we compared it to existing connectivity matrices generated from two other brain mapping modalities: CCEP and rs-fMRI correlation magnitude. As shown Figure 9*A*, the qualitative pattern of rs-fMRI markedly differs from the other two modalities with proportionally stronger ipsilateral across-network connections and especially non-homologous contralateral connections, although the latter is somewhat obscured for CCEPs because of sparse spatial sampling. Over all connections, pairwise probabilistic dMRI connectivity values are nearly twice as linearly correlated to pairwise CCEP connectivity than to rs-fMRI connectivity (Fig. 9*B*), and this contrast is equally evident in the ipsilateral connection within each hemisphere (Extended Data Fig. 9-1). Contralateral connections were not examined in isolation as contralateral sampling for the CCEP modality is relatively rare.

10.1523/ENEURO.0416-20.2020.f9-1 Extended Data Figure 9-1 Within-hemisphere comparison of probabilistic dMRI tractography, CCEP, and rs-fMRI connectivity. For the left and right hemisphere, the distribution of pairwise non-zero connection strengths and correlations among the three modalities are shown. The least-squares linear fit is shown in red. All within-hemisphere findings are concordant with the overall findings, shown in Figure 9. Download Figure 9-1, TIF file.

When comparing the distributions of pairwise connectivity strength (Fig. 9*C*), rs-fMRI again exhibits properties different from the other two modalities. While both dMRI and CCEP distributions skew in opposite directions (0.63 and −0.43, respectively), their strengths form unimodal log-normal distributions and thus shown with log-transformed values. In contrast, rs-fMRI connectivity values form a bimodal Gaussian-mixture distribution in linear space. The two modes were characterized by obtaining the maximum-likelihood fit (fitgmdist) of a two-component Gaussian-mixture to the data, yielding a left mode (μ = 0.0011, σ = 8.1e-8) forming 63% of the distribution and a right mode (μ = 0.0017, σ = 8.1e-8) forming 37%, respectively. Splitting the rs-fMRI modes at the midpoint between their means (0.0014) and plotting their respective connectivity matrices (Fig. 9*D*) reveals that the low-connectivity (left) mode consists primarily of connections between the default mode/frontoparietal networks and other regions of the cortex.

**Figure 10.**
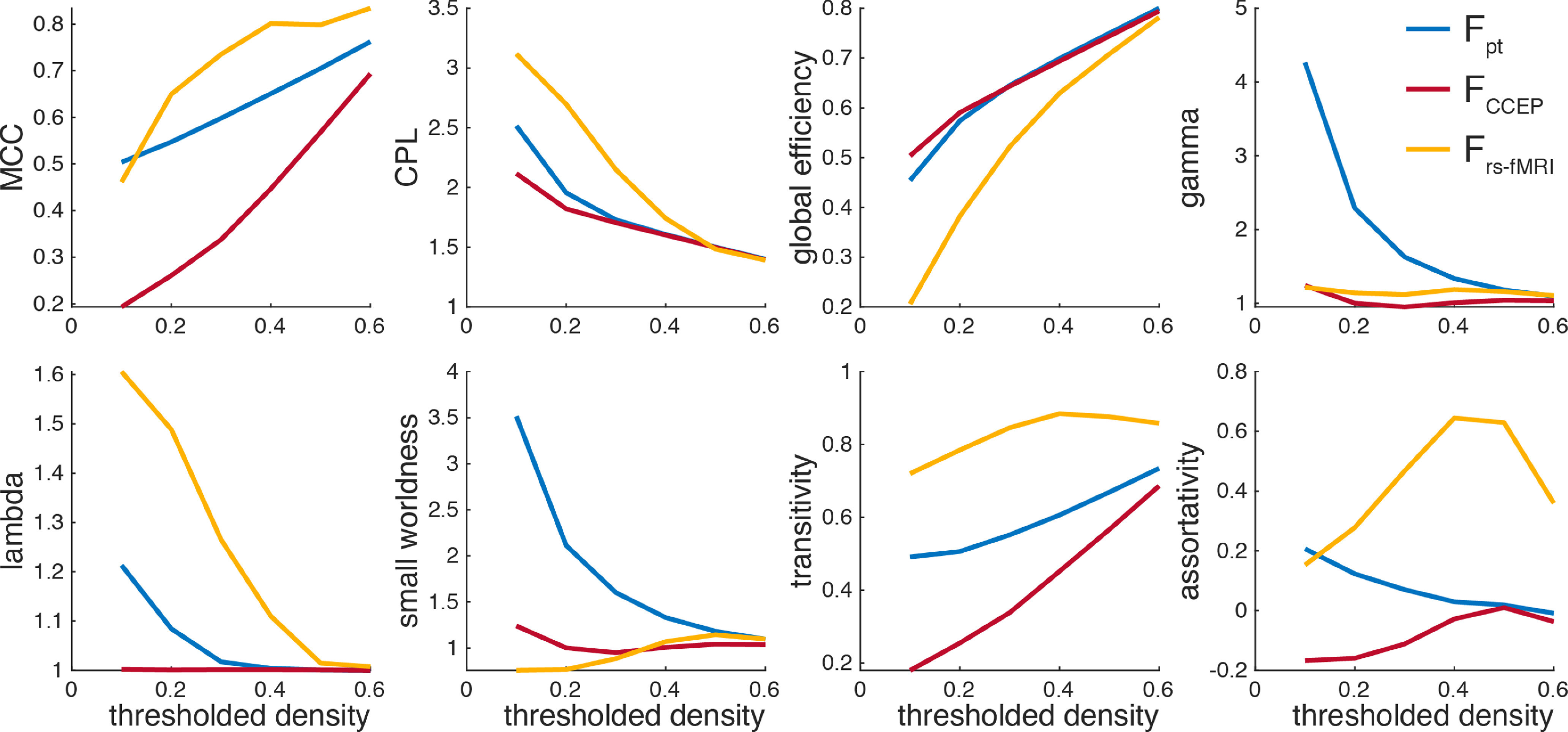
Network theoretic differences between the connectivity modalities. Binarized network metrics after thresholding by edge weight (connectivity strength).

To further contrast the three connectivity modalities, we computed six network theoretic metrics for each of the connectivity matrices: MCC, CPL, global efficiency, γ (normalized MCC), λ (normalized CPL), small-worldness, transitivity, and assortativity (see Eq. 3–18). Binarized network metrics were assessed after thresholding by edge weight (connectivity strength) at intervals of 0.1. Note that this λ is unrelated to the exponential length constant reported above. To account for the order-based arbitrary treatment of equal edge weights when thresholding, the node (parcel) order was randomized 1000 times, and the mean metric values are shown. Empirical 95% confidence intervals for these means are too small to be shown at scale. Networks densities above 0.6 were not examined as the un-thresholded network density of CCEP connectivity matrix, treating missing data as non-connections, is <0.7. However, all measures appear to converge as binary network density approaches 1. As shown in Figure 10, the MCC, CPL, global efficiency, small-worldness, transitivity, and assortativity are markedly different for rs-fMRI connectivity than for CCEP and probabilistic dMRI tractography, whose metrics as a function of network density are more similar to each other. Normalizing by metrics computed for a random network with the same statistical makeup changes this pattern. For γ, the rs-fMRI and CCEP networks are more similar than either is to probabilistic dMRI tractography, and λ rs-fMRI and probabilistic dMRI tractography are more similar than either is to the CCEP network. The high MCC, transitivity, and assortativity and low global efficiency of rs-fMRI relative to the other modalities may be indicative of strong, long-range correlativity beyond that predicted by anatomic connections.

## Discussion

In this study we compiled a whole-cortex structural connectome by applying probabilistic tractography to the diffusion MR volumes of 1065 subjects from the WU-Minn HCP. We report a novel, complete, and high-dynamic-range connectivity matrix discretized into the 360 parcels of the HCP-MMP1.0 atlas and further arranged into 10 functional networks. It is shown that connectivity strength exponentially decays with fiber tract length, that the parts of the connectome with clear homology to macaques correspond reasonably to retrograde tracer mappings in that species, that contralateral homologs are hyperconnected, and that some connections within language-implicated cortex are stronger than expected and left-lateralized. While ipsilateral connectivity generally dominates, some regions have stronger contralateral connections. Interindividual variability is relatively high for early visual cortex, whose connectivity co-varies across hemispheres. Cortical areas tend to be more connected with areas at similar levels of the cortical hierarchy, as indexed by their estimated myelination, particularly in prefrontal areas. Lastly, it is shown that probabilistic tractography connectivity more closely resembles that of CCEPs than rs-fMRI. In sum, we quantify a dMRI-based estimate of medium-range to long-range anatomic corticocortical connectivity in a large normative sample.

dMRI and automated *post hoc* tractography are powerful tools for the elucidation of cerebral connectivity. The defining advantages of these techniques are non-invasiveness and large field-of-view, enabling whole-brain mapping in humans. However, dMRI does have significant limitations when compared with histological fiber tracing, electron microscopy, or stimulation. The most obvious of these is insensitivity to whether underlying axons are anterograde or retrograde, as evidenced by the symmetry of the connectivity matrix. The anisotropic diffusion of water molecules occurs in both anterograde and retrograde directions. Thus, the true one-way connectivity between two areas could be anywhere between none to all of the symmetric diffusion connectivity. Another important limitation is spatial resolution. While the 1.25-mm isotropic voxels achieved by the WU-Minn dMRI protocol are smaller than those of most studies (Jeurissen et al., 2019), they are still more than three orders-of-magnitude larger than the typical submicron axon diameter (von Keyserlingk Graf and Schramm, 1984; Liewald et al., 2014). This discrepancy is particularly impactful when fiber orientations are not consistent within a voxel, i.e., crossing fibers. Probabilistic diffusion tractography (Behrens et al., 2007) partially ameliorates the issue by modeling the probability distribution of orientations and accounting for uncertainty, but ultimately dMRI with current technology is a meso- to macroscale technique. Direct histologic validation of dMRI techniques is uncommon, but has been performed for probabilistic tractography *in vitro* in pigs (Dyrby et al., 2007) and macaques (Jbabdi et al., 2013; Donahue et al., 2016), with the latter two studies using the same probtrackX algorithm as the current study (Behrens et al., 2007). We have extended these validations with a between-species comparison (Fig. 4).

Of the several families of dMRI tractography algorithms available, we selected local, probabilistic tractography (Behrens et al., 2007). The WU-Minn HCP makes available the bedpostX precursor files and creating a probabilistic tractography connectome was always a stated component of the WU-Minn HCP project (Van Essen et al., 2013; Van Essen and Ugurbil, 2017). That such a connectome has not yet been released for these data may be because of the immense computational challenge of performing these analyses at the scale of the HCP. An advantage of probabilistic tractography is its sensitivity to minor, or low-probability connections. Deterministic dMRI tractography connectomes typically have low network densities, e.g., 0.18 (Mori et al., 2008) or 0.23 (Cui et al., 2019), when compared with histologic fiber tracing in macaques, 0.66 (Markov et al., 2014), and this is likely a lower bound as such tracing is subject to false-negatives because of imperfect dye uptake and incomplete cortical sampling. This suggests the deterministic dMRI connectomes are missing weaker connections. On the other hand, dMRI in general and probabilistic tractography in particular has been found vulnerable to false-positive connections (Maier-Hein et al., 2017). This exchange of specificity for sensitivity (Zalesky et al., 2016; Sarwar et al., 2019) is consistent with our very high group-average network density of 1.0 and the likely presence false-positive connections and is, thus, an important caveat to the data presented here. In cases where false-negative connections are less concerning than false-positive connections, such as topological analyses (Zalesky et al., 2016), subsequent users of these data may opt to threshold the connectivity matrix by either connection strength or consistency (Fig. 3; Roberts et al., 2017).

When constructing this connectome, we divided the cortex into 180 parcels per hemisphere following the HCP-MMP1.0 atlas (Glasser et al., 2016). To ease interpretation, we further organized the parcels into 10 functional networks modified from (Ji et al., 2019). These networks were created by applying iterative Louvain clustering (Blondel et al., 2008; Rubinov and Sporns, 2010) and other criteria to HCP rs-fMRI data. While these fMRI-defined network definitions correspond reasonably to the structural connections reported here, there are exceptions. The operculum and temporoparietal junction, in particular, appears to be a structurally distinct area that has been folded into several functional networks (Ji et al., 2019). However, this contiguous region forms the lateral salience network in Barnett et al. (2020), which similarly applied a very similar methodology to a non-HCP cohort. Like many cortically-focused studies, we used a surface-based methodology to define these areas, with seed and target regions constrained to the white-matter–gray-matter interface. This approach reduces the overrepresentation of major bundles (Jeurissen et al., 2019), enables the automated assessment based on intersubject homology (Fischl et al., 1999), facilitates comparison to other cortical datasets, and is true to the anatomic nature of the cortical ribbon. Unfortunately, the subcortex and cerebellum are omitted in this analysis, as are short-range, often unmyelinated, intraparcel connections. While the inclusion of the thalamic radiations, in particular, is a merited future extension of this connectome, the small size of subcortical structures relative to diffusion imaging voxels, the nuclear (as opposed the sheet-like) organization of subcortical structures, and complex geometry of the subcortical white matter, gray matter interface (e.g., the internal medullary lamina of the thalamus), all render the challenges and methods for obtaining subcortical tractography substantially distinct from those of cortico-cortico tractography.

The HCP-MMP1.0 atlas used was selected because of its wide adoption, symmetry, and high parcel count. Furthermore, the parcels are based on multiple functional and anatomic criteria and are consistent with previous functional parcellations in human and non-human primates (Felleman and Van Essen, 1991; Glasser et al., 2016). Because the parcels are relatively small and informed by function, erroneous averaging of disparate connections, a connectomic extension of the partial volume artifact, is minimized. However, this comes at the cost of non-uniformity in both parcel area and shape. Methodologically, parcels are assembled from vertices on the tessellated cortical surface. A future vertex-based or voxel-based connectome, while computationally challenging, would have the distinct advantage of being readily reformulated into any arbitrary surface-based parcellation scheme.

We found that pairwise connectivity between cortical parcels exhibits an exponential decay rule with respect to fiber tract distance with a length constant λ of ∼23 mm (∼33 mm for callosal connections). While a tight exponential relationship between probabilistic diffusion tractography strength and fiber length has been previously reported (Roberts et al., 2016), this study did not report the observed λ or release its data. Histologic studies in non-human primates (Markov et al., 2013; Donahue et al., 2016; Theodoni et al., 2020) consistently show exponential connectivity decay with distance. Such a rule when combined with a roughly Gaussian distribution of interareal distances explains the observed log-normal distribution of connectivity strength (Markov et al., 2013). Histologic data indicate a λ of ∼3.33 mm for marmosets (Theodoni et al., 2020) and 5.55 mm for macaques (Markov et al., 2013). Across species, there appears to be a linear relationship between the logs of λ and total gray matter volume, predicting a human λ of 10 mm (Theodoni et al., 2020). While methodological differences between diffusion and histologic tractography cannot be completely ruled out, Donahue and colleagues found similar λ for the two methods in macaques (Donahue et al., 2016). Our results suggest that, compared with other species, human cortical areas are exceptionally well connected relative to their cortical volume, reflected in a disproportionately long λ. Conservatively restricting the exponential fit to only the most consistent quintile of connections (Fig. 3*D*) yields a λ of ∼28 mm, further accentuating the proportional long-range hyperconnectivity of humans.

Geometric scaling strongly constrains corticocortical connectivity in humans. Considering primate brains increasing in diameter d, volume and number of cortical neurons increases by d^3^ (Ventura-Antunes et al., 2013), so arriving at a constant probability of connection between any two neurons would require d^6^ axons, and since they would need to be about d times as long, this would require a volume proportional to d^7^, or more if axonal diameter is increased to maintain a relatively constant latency of communication (Wang et al., 2008). However, the actual white matter volume is less than d^4^ (Zhang and Sejnowski, 2000), and consequently the probability of corticocortical connectivity must be highly limited in humans. The relatively long λ in humans we report reduces even further the number of connections which can be accommodated within the available white matter volume. A consequence of fewer but longer connections would be reduced metabolic cost, inasmuch the cost of an action potential is 1/3 axonal transmission (proportional to length) and 2/3 synaptic transmission (Lennie, 2003). The low firing rate of human pyramidal cells (Chan et al., 2014) would also reduce the metabolic cost of their axons. These observations are consistent with the proposal that the metabolic costs of corticocortical connections may help constrain their organization in the primate brain (Ercsey-Ravasz et al., 2013). Given this strong correlation of connection strength with distance, as well as the bias of tract-tracing techniques toward shorter, less geometrically complex connections (Jeurissen et al., 2019), there may be some merit in regressing out the effect of tract length when evaluating the relative connectivity of different cortical areas. However, the considerations enumerated above imply a strong evolutionary selection to place cortical parcels which require high connectivity to perform their calculations to be situated in direct physical proximity to each other. The patterns of relatively long-distance connectivity identified here thus must be viewed as minor deviations from an overall strong tendency favoring local connectivity, a conceptualization consistent with the view of the cortex as a spatially embedded small world network.

One striking deviation from the distance-based connectivity was the left-lateralized hyperconnectivity between language areas, and specifically between posterior and anterior language areas. This connectivity presumably passes, completely or in part, through the classical language pathways (for review, see Dick and Tremblay, 2012). The lateralization we observed may then reflect that of the arcuate and inferior longitudinal fasciculi which connect the same structures and show significant left lateralization in humans but not macaques (Panesar et al., 2018; Eichert et al., 2019). Left-lateralization of the arcuate fasciculus develops late (Lebel and Beaulieu, 2011), and is sensitive to the presence, quality and quantity of early language experience (Romeo et al., 2018; Cheng et al., 2019). More generally, many of the connectivity patterns observed here could be the indirect result of co-activation of the connected parcels (Mount and Monje, 2017). The left-lateralized ipsilateral connectivity may be compensated by a relative lack of callosal connections from the same areas, under the hypothesis that the total connectivity is constrained.

A more general factor that might induce deviations from a distance-based connectivity rule may be the principle of hierarchical organization. It has been proposed that distant areas with similar laminar properties, and thus of similar hierarchical order may have privileged connections (Barbas, 2015). Across the entire cortex we find that myelination similarity explains a significant but small amount of the overall variance. However, there are regions where the influence of hierarchical position is more pronounced including the right dorsal attention and left auditory/language networks. The observed hyperconnectivity and high degree of lateralization in these regions may be a consequence of the low-latencies necessary for the functions they underly. More broadly, the effects of transmission latency constraints on neuroanatomy and conduction delay on large-scale physiological recordings are an emerging area of study in human neuroscience (Muller et al., 2018). Latency is a hybrid structural–functional property of connectivity, and might in future be quantified using the latency of CCEPs.

By emphasizing the cortical connectivity matrix over the white matter bundles per se and organizing the matrix into the widely adopted HCP-MMP1.0 atlas (Glasser et al., 2016), the structural connectome reported here enables ready comparison to other structural, functional, and hybrid connectomes. As an example, we compared the probabilistic tractography connectivity to exist rs-fMRI (Van Essen et al., 2013) and CCEP (Trebaul et al., 2018) connectivity matrices and found that our dMRI-inferred structural connectivity better reflects CCEP probability than rs-fMRI connectivity in both linear and network-theoretic comparisons, despite the dMRI and rs-fMRI cohorts being highly overlapping. This is not unreasonable, as functional correlations are to varying degrees neurobehavioral state dependent and far more spatiotemporally dynamic than structural connections. Furthermore, although resting-state functional connectivity is constrained by anatomic networks and can be partially predicted by them (Honey et al., 2009), indirect connections or parallel processing of stimuli in different areas can produce correlated activity even in the absence of direct anatomic connections. One notable example of the latter may be interhemispheric connectivity. While we did find hyperconnectivity between interhemispheric homologs when compared with other callosal connections, anatomic interhemispheric connectivity on the whole is much weaker than found in rs-fMRI. CCEPs, being directed by clinical requirements, have poor interhemispheric sampling, but we found that even among ipsilateral connections, rs-fMRI is still less similar to CCEP than probabilistic tractography. These intermodal connectivity comparisons are not intended to be comprehensive. The HCP cohort also includes source-localized resting-state MEG (Larson-Prior et al., 2013), which could be used to examine the degree to which the functional connectivity of various frequency bands corresponds to anatomic connectivity. Furthermore, neuropsychological metrics, including the NIH toolbox (Gershon et al., 2013), and genotypic data (dbGaP phs001364.v1.p1) are also available for this cohort, enabling future studies of the interplay between cortical connectivity, cognition, and genetics.

The HCP was a scientific undertaking of visionary scope and ambition. Its commitment to open science and accessibility of data by the public enabled this study and will continue to facilitate further studies for years to come. Emerging clinical applications of brain connectomics will be underpinned by a strong base of normative data for comparison. The whole-cortex probabilistic diffusion tractography connectome reported here fulfills a key goal outlined in the project’s conception and we hope it will empower yet further study of the myriad and beautiful web of connectivity that the human brain embodies.

## Synthesis

Reviewing Editor: Gustavo Deco, Universitat Pompeu Fabra Decisions are customarily a result of the Reviewing Editor and the peer reviewers coming together and discussing their recommendations until a consensus is reached. When revisions are invited, a fact-based synthesis statement explaining their decision and outlining what is needed to prepare a revision will be listed below. The following reviewer(s) agreed to reveal their identity: Christian Márton.

The paper provides insight into the human brain by presenting a structural connectome based on probabilistic diffusion tractography. Its main novelty aspect are the large number of subjects and the suitable parcellation scheme used, that facilitate in making such a structural connectome useful to the neuroscience community. On top of that, the additional validation analyses performed ensure its reliability and robustness.

## References

[B1] Andersson JLR, Sotiropoulos SN (2016) An integrated approach to correction for off-resonance effects and subject movement in diffusion MR imaging. Neuroimage 125:1063–1078. 10.1016/j.neuroimage.2015.10.019 26481672 PMC4692656

[B2] Barbas H (2015) General cortical and special prefrontal connections: principles from structure to function. Annu Rev Neurosci 38:269–289. 10.1146/annurev-neuro-071714-033936 25897871

[B3] Barnett AJ, Reilly W, Dimsdale-Zucker H, Mizrak E, Reagh Z, Ranganath C (2020) Organization of cortico-hippocampal networks in the human brain bioRxiv. doi: 10.1101/2020.06.09.142166. PMC8202937 34077415

[B4] Behrens TEJ, Berg HJ, Jbabdi S, Rushworth MFS, Woolrich MW (2007) Probabilistic diffusion tractography with multiple fibre orientations: what can we gain? Neuroimage 34:144–155. 10.1016/j.neuroimage.2006.09.018 17070705 PMC7116582

[B5] Blondel VD, Guillaume JL, Lambiotte R, Lefebvre E (2008) Fast unfolding of communities in large networks. J Stat Mech Theory Exp 10:P10008.

[B6] Burns S (2014) A recipe for cortical tractography using Freesufer labels. Available from http://sburns.org/2014/05/03/cortical-tractography-recipe.html.

[B7] Burt JB, Demirtaş M, Eckner WJ, Navejar NM, Ji JL, Martin WJ, Bernacchia A, Anticevic A, Murray JD (2018) Hierarchy of transcriptomic specialization across human cortex captured by structural neuroimaging topography. Nat Neurosci 21:1251–1259. 10.1038/s41593-018-0195-0 30082915 PMC6119093

[B8] Chan AM, Dykstra AR, Jayaram V, Leonard MK, Travis KE, Gygi B, Baker JM, Eskandar E, Hochberg LR, Halgren E, Cash SS (2014) Speech-specific tuning of neurons in human superior temporal gyrus. Cereb Cortex 24:2679–2693. 10.1093/cercor/bht127 23680841 PMC4162511

[B9] Cheng Q, Roth A, Halgren E, Mayberry RI (2019) Effects of early language deprivation on brain connectivity: language pathways in deaf native and late first-language learners of American sign language. Front Hum Neurosci 13:1–12. 31607879 10.3389/fnhum.2019.00320 PMC6761297

[B10] Coalson T, Van Essen D, Glasser M (2016) hcp-users FAQ #9: how do I map data between FreeSurfer and HCP? Available at https://wiki.humanconnectome.org/download/attachments/63078513/Resampling-FreeSurfer-HCP.pdf.

[B11] Cui LB, Wei Y, Xi Y, Bin Griffa A, De Lange SC, Kahn RS, Yin H, Van Den Heuvel MP (2019) Connectome-based patterns of first-episode medication-naïve patients with schizophrenia. Schizophr Bull 45:1291–1299. 10.1093/schbul/sbz014 30926985 PMC6811827

[B12] David O, Job AS, De Palma L, Hoffmann D, Minotti L, Kahane P (2013) Probabilistic functional tractography of the human cortex. Neuroimage 80:307–317. 10.1016/j.neuroimage.2013.05.075 23707583

[B13] Dayan P, Abbott LF (2001) Theoretical neuroscience: computational and mathematical modeling of neural systems. Cambridge: The MIT Press.

[B14] Desikan RS, Ségonne F, Fischl B, Quinn BT, Dickerson BC, Blacker D, Buckner RL, Dale AM, Maguire RP, Hyman BT, Albert MS, Killiany RJ (2006) An automated labeling system for subdividing the human cerebral cortex on MRI scans into gyral based regions of interest. Neuroimage 31:968–980. 10.1016/j.neuroimage.2006.01.021 16530430

[B15] Dick AS, Tremblay P (2012) Beyond the arcuate fasciculus: consensus and controversy in the connectional anatomy of language. Brain 135:3529–3550. 10.1093/brain/aws222 23107648

[B16] Donahue CJ, Sotiropoulos SN, Jbabdi S, Hernandez-Fernandez M, Behrens TE, Dyrby TB, Coalson T, Kennedy H, Knoblauch K, Van Essen DC, Glasser MF (2016) Using diffusion tractography to predict cortical connection strength and distance: a quantitative comparison with tracers in the monkey. J Neurosci 36:6758–6770. 10.1523/JNEUROSCI.0493-16.2016 27335406 PMC4916250

[B17] Dyrby TB, Søgaard LV, Parker GJ, Alexander DC, Lind NM, Baaré WFC, Hay-Schmidt A, Eriksen N, Pakkenberg B, Paulson OB, Jelsing J (2007) Validation of in vitro probabilistic tractography. Neuroimage 37:1267–1277. 10.1016/j.neuroimage.2007.06.022 17706434

[B18] Eichert N, Verhagen L, Folloni D, Jbabdi S, Khrapitchev AA, Sibson NR, Mantini D, Sallet J, Mars RB (2019) What is special about the human arcuate fasciculus? Lateralization, projections, and expansion. Cortex 118:107–115. 10.1016/j.cortex.2018.05.005 29937266 PMC6699597

[B19] Ercsey-Ravasz M, Markov NT, Lamy C, Van Essen DC, Knoblauch K, Toroczkai Z, Kennedy H (2013) A predictive network model of cerebral cortical connectivity based on a distance rule. Neuron 80:184–197. 10.1016/j.neuron.2013.07.036 24094111 PMC3954498

[B20] Felleman DJ, Van Essen DC (1991) Distributed hierarchical processing in the primate cerebral cortex. Cereb Cortex 1:1–47. 10.1093/cercor/1.1.1 1822724

[B21] Fischl B (2012) FreeSurfer. Neuroimage 62:774–781. 10.1016/j.neuroimage.2012.01.021 22248573 PMC3685476

[B22] Fischl B, Sereno MI, Tootell RBH, Dale AM (1999) High-resolution intersubject averaging and a coordinate system for the cortical surface. Hum Brain Mapp 8:272–284. 10.1002/(SICI)1097-0193(1999)8:4<272::AID-HBM10>3.0.CO;2-4 10619420 PMC6873338

[B23] Fischl B, Van Der Kouwe A, Destrieux C, Halgren E, Ségonne F, Salat DH, Busa E, Seidman LJ, Goldstein J, Kennedy D, Caviness V, Makris N, Rosen B, Dale AM (2004) Automatically parcellating the human cerebral cortex. Cereb Cortex 14:11–22. 10.1093/cercor/bhg087 14654453

[B24] Gershon RC, Wagster MV, Hendrie HC, Fox NA, Cook KF, Nowinski CJ (2013) NIH toolbox for assessment of neurological and behavioral function. Neurology 80:S2–S6. 10.1212/WNL.0b013e3182872e5f 23479538 PMC3662335

[B25] Glasser MF, Van Essen DC (2011) Mapping human cortical areas in vivo based on myelin content as revealed by T1- and T2-weighted MRI. J Neurosci 31:11597–11616. 10.1523/JNEUROSCI.2180-11.2011 21832190 PMC3167149

[B26] Glasser MF, Coalson TS, Robinson EC, Hacker CD, Harwell J, Yacoub E, Ugurbil K, Andersson J, Beckmann CF, Jenkinson M, Smith SM, Van Essen DC (2016) A multi-modal parcellation of human cerebral cortex. Nature 536:171–178. 10.1038/nature18933 27437579 PMC4990127

[B27] Hagmann P, Cammoun L, Gigandet X, Meuli R, Honey CJ, Van Wedeen J, Sporns O (2008) Mapping the structural core of human cerebral cortex. PLoS Biol 6:e159. 10.1371/journal.pbio.0060159 18597554 PMC2443193

[B28] Honey CJ, Sporns O, Cammoun L, Gigandet X, Thiran JP, Meuli R, Hagmann P (2009) Predicting human resting-state functional connectivity from structural connectivity. Proc Natl Acad Sci USA 106:2035–2040. 10.1073/pnas.0811168106 19188601 PMC2634800

[B29] Humphries MD, Gurney K (2008) Network “small-world-ness”: a quantitative method for determining canonical network equivalence. PLoS One 3:e0002051. 10.1371/journal.pone.0002051 18446219 PMC2323569

[B30] Jbabdi S, Lehman JF, Haber SN, Behrens TE (2013) Human and monkey ventral prefrontal fibers use the same organizational principles to reach their targets: tracing versus tractography. J Neurosci 33:3190–3201. 10.1523/JNEUROSCI.2457-12.2013 23407972 PMC3602794

[B31] Jenkinson M, Beckmann CF, Behrens TEJ, Woolrich MW, Smith SM (2012) FSL. Neuroimage 62:782–790. 10.1016/j.neuroimage.2011.09.015 21979382

[B32] Jeurissen B, Descoteaux M, Mori S, Leemans A (2019) Diffusion MRI fiber tractography of the brain. NMR Biomed 32:1–22. 10.1002/nbm.3785 28945294

[B33] Ji JL, Spronk M, Kulkarni K, Repovš G, Anticevic A, Cole MW (2019) Mapping the human brain’s cortical-subcortical functional network organization. Neuroimage 185:35–57. 10.1016/j.neuroimage.2018.10.006 30291974 PMC6289683

[B34] Larson-Prior LJ, Oostenveld R, Della Penna S, Michalareas G, Prior F, Babajani-Feremi A, Schoffelen JM, Marzetti L, de Pasquale F, Di Pompeo F, Stout J, Woolrich M, Luo Q, Bucholz R, Fries P, Pizzella V, Romani GL, Corbetta M, Snyder AZ, et al. (2013) Adding dynamics to the Human Connectome Project with MEG. Neuroimage 80:190–201. 10.1016/j.neuroimage.2013.05.056 23702419 PMC3784249

[B35] Latora V, Marchiori M (2001) Efficient behavior of small-world networks. Phys Rev Lett 87:198701. 11690461 10.1103/PhysRevLett.87.198701

[B36] Lebel C, Beaulieu C (2011) Longitudinal development of human brain wiring continues from childhood into adulthood. J Neurosci 31:10937–10947. 10.1523/JNEUROSCI.5302-10.2011 21795544 PMC6623097

[B37] Lennie P (2003) The cost of cortical computation. Curr Biol 13:493–497. 10.1016/s0960-9822(03)00135-0 12646132

[B38] Liewald D, Miller R, Logothetis N, Wagner HJ, Schüz A (2014) Distribution of axon diameters in cortical white matter: an electron-microscopic study on three human brains and a macaque. Biol Cybern 108:541–557. 10.1007/s00422-014-0626-2 25142940 PMC4228120

[B39] Maier-Hein KH, Neher PF, Houde JC, Côté MA, Garyfallidis E, Zhong J, Chamberland M, Yeh FC, Lin YC, Ji Q, Reddick WE, Glass JO, Chen DQ, Feng Y, Gao C, Wu Y, Ma J, Renjie H, Li Q, Westin CF, et al. (2017) The challenge of mapping the human connectome based on diffusion tractography. Nat Commun 8:1349. 10.1038/s41467-017-01285-x 29116093 PMC5677006

[B40] Marcus DS, Harwell J, Olsen T, Hodge M, Glasser MF, Prior F, Jenkinson M, Laumann T, Curtiss SW, Van Essen DC (2011) Informatics and data mining tools and strategies for the human connectome project. Front Neuroinform 5:1–12. 21743807 10.3389/fninf.2011.00004 PMC3127103

[B41] Markov NT, Ercsey-Ravasz M, Van Essen DC, Knoblauch K, Toroczkai Z, Kennedy H (2013) Cortical high-density counterstream architectures. Science 342:1238406. 10.1126/science.1238406 24179228 PMC3905047

[B42] Markov NT, Ercsey-Ravasz MM, Ribeiro Gomes AR, Lamy C, Magrou L, Vezoli J, Misery P, Falchier A, Quilodran R, Gariel MA, Sallet J, Gamanut R, Huissoud C, Clavagnier S, Giroud P, Sappey-Marinier D, Barone P, Dehay C, Toroczkai Z, et al. (2014) A weighted and directed interareal connectivity matrix for macaque cerebral cortex. Cereb cortex 24:17–36. 10.1093/cercor/bhs270 23010748 PMC3862262

[B43] Mori S, Oishi K, Jiang H, Jiang L, Li X, Akhter K, Hua K, Faria AV, Mahmood A, Woods R, Toga AW, Pike GB, Neto PR, Evans A, Zhang J, Huang H, Miller MI, van Zijl P, Mazziotta J (2008) Stereotaxic white matter atlas based on diffusion tensor imaging in an ICBM template. Neuroimage 40:570–582. 10.1016/j.neuroimage.2007.12.035 18255316 PMC2478641

[B44] Mount CW, Monje M (2017) Wrapped to adapt: experience-dependent myelination. Neuron 95:743–756. 10.1016/j.neuron.2017.07.009 28817797 PMC5667660

[B45] Muller L, Chavane F, Reynolds J, Sejnowski TJ (2018) Cortical travelling waves: mechanisms and computational principles. Nat Rev Neurosci 19:255–268. 10.1038/nrn.2018.20 29563572 PMC5933075

[B46] Newman MEJ (2003) The structure and function of complex networks. SIAM Rev 45:167–256. 10.1137/S003614450342480

[B47] Newman MEJ (2004) Fast algorithm for detecting community structure in networks. Phys Rev E 69:66133. 10.1103/PhysRevE.69.066133 15244693

[B48] Panesar SS, Yeh FC, Jacquesson T, Hula W, Fernandez-Miranda JC (2018) A quantitative tractography study into the connectivity, segmentation and laterality of the human inferior longitudinal fasciculus. Front Neuroanat 12:47. 10.3389/fnana.2018.00047 29922132 PMC5996125

[B49] Roberts JA, Perry A, Lord AR, Roberts G, Mitchell PB, Smith RE, Calamante F, Breakspear M (2016) The contribution of geometry to the human connectome. Neuroimage 124:379–393. 10.1016/j.neuroimage.2015.09.009 26364864

[B50] Roberts JA, Perry A, Roberts G, Mitchell PB, Breakspear M (2017) Consistency-based thresholding of the human connectome. Neuroimage 145:118–129. 10.1016/j.neuroimage.2016.09.053 27666386

[B51] Romeo RR, Segaran J, Leonard JA, Robinson ST, West MR, Mackey AP, Yendiki A, Rowe ML, Gabrieli JDE (2018) Language exposure relates to structural neural connectivity in childhood. J Neurosci 38:7870–7877. 10.1523/JNEUROSCI.0484-18.2018 30104336 PMC6125810

[B52] Rubinov M, Sporns O (2010) Complex network measures of brain connectivity: uses and interpretations. Neuroimage 52:1059–1069. 10.1016/j.neuroimage.2009.10.003 19819337

[B53] Sarwar T, Ramamohanarao K, Zalesky A (2019) Mapping connectomes with diffusion MRI: deterministic or probabilistic tractography? Magn Reson Med 81:1368–1384. 10.1002/mrm.27471 30303550

[B54] Sotiropoulos SN, Jbabdi S, Xu J, Andersson JL, Moeller S, Auerbach EJ, Glasser MF, Hernandez M, Sapiro G, Jenkinson M, Feinberg DA, Yacoub E, Lenglet C, Van Essen DC, Ugurbil K, Behrens TEJ; WU-Minn HCP Consortium (2013) Advances in diffusion MRI acquisition and processing in the Human Connectome Project. Neuroimage 80:125–143. 10.1016/j.neuroimage.2013.05.057 23702418 PMC3720790

[B56] Theodoni P, Majka P, Reser DH, Wójcik DK, Rosa MGP, Wang XJ (2020) Structural attributes and principles of the neocortical connectome in the marmoset monkey. bioRxiv. doi: 10.1101/2020.02.28.969824. PMC8634603 34274966

[B57] Trebaul L, Deman P, Tuyisenge V, Jedynak M, Hugues E, Rudrauf D, Bhattacharjee M, Tadel F, Chanteloup-Foret B, Saubat C, Reyes Mejia GC, Adam C, Nica A, Pail M, Dubeau F, Rheims S, Trébuchon A, Wang H, Liu S, et al. (2018) Probabilistic functional tractography of the human cortex revisited. Neuroimage 181:414–429. 10.1016/j.neuroimage.2018.07.039 30025851 PMC6150949

[B58] Van Essen DC, Ugurbil K (2017) Components of the Human Connectome Project - diffusion tractography. Available from https://www.humanconnectome.org/study/hcp-young-adult/project-protocol/diffusion-tractography.

[B59] Van Essen DC, Smith SM, Barch DM, Behrens TEJ, Yacoub E, Ugurbil K; WU-Minn HCP Consortium (2013) The WU-Minn Human Connectome Project: an overview. Neuroimage 80:62–79. 10.1016/j.neuroimage.2013.05.041 23684880 PMC3724347

[B60] Ventura-Antunes L, Mota B, Herculano-Houzel S (2013) Different scaling of white matter volume, cortical connectivity, and gyrification across rodent and primate brains. Front Neuroanat 7:3–12. 10.3389/fnana.2013.00003 23576961 PMC3620553

[B61] von Keyserlingk Graf D, Schramm U (1984) Diameter of axons and thickness of myelin sheaths of the pyramidal tract fibres in the adult human medullary pyramid. Anat Anz 157:97–111. 6507887

[B62] Wang SSH, Shultz JR, Burish MJ, Harrison KH, Hof PR, Towns LC, Wagers MW, Wyatt KD (2008) Functional trade-offs in white matter axonal scaling. J Neurosci 28:4047–4056. 10.1523/JNEUROSCI.5559-05.2008 18400904 PMC2779774

[B63] Watts DJ, Strogatz SH (1998) Collective dynamics of ‘small-world’ networks. Nature 393:440–442. 10.1038/30918 9623998

[B64] Yeh FC, Verstynen TD, Wang Y, Fernández-Miranda JC, Tseng WYI (2013) Deterministic diffusion fiber tracking improved by quantitative anisotropy. PLoS One 8:e80713. 10.1371/journal.pone.0080713 24348913 PMC3858183

[B65] Yeh FC, Panesar S, Fernandes D, Meola A, Yoshino M, Fernandez-Miranda JC, Vettel JM, Verstynen T (2018) Population-averaged atlas of the macroscale human structural connectome and its network topology. Neuroimage 178:57–68. 10.1016/j.neuroimage.2018.05.027 29758339 PMC6921501

[B67] Yoon JM, Benson NC, Forenzo D, Winawer J, Engel SA, Kay KN (2019) Heritability of V1/V2/V3 surface area in the HCP 7T Retinotopy Dataset. J Vis 19:41b. 10.1167/19.10.41b

[B68] Zalesky A, Fornito A, Cocchi L, Gollo LL, van den Heuvel MP, Breakspear M (2016) Connectome sensitivity or specificity: which is more important? Neuroimage 142:407–420. 10.1016/j.neuroimage.2016.06.035 27364472

[B69] Zhang K, Sejnowski TJ (2000) A universal scaling law between gray matter and white matter of cerebral cortex. Proc Natl Acad Sci USA 97:5621–5626. 10.1073/pnas.090504197 10792049 PMC25878

