# Supplementary figures and images for: A Whole-Cortex Probabilistic Diffusion Tractography Connectome
Source: eNeuro. 2021 Feb 2;8(1):ENEURO.0416-20.2020. doi: 10.1523/ENEURO.0416-20.2020 (PMC7920542; doi:10.1523/ENEURO.0416-20.2020)

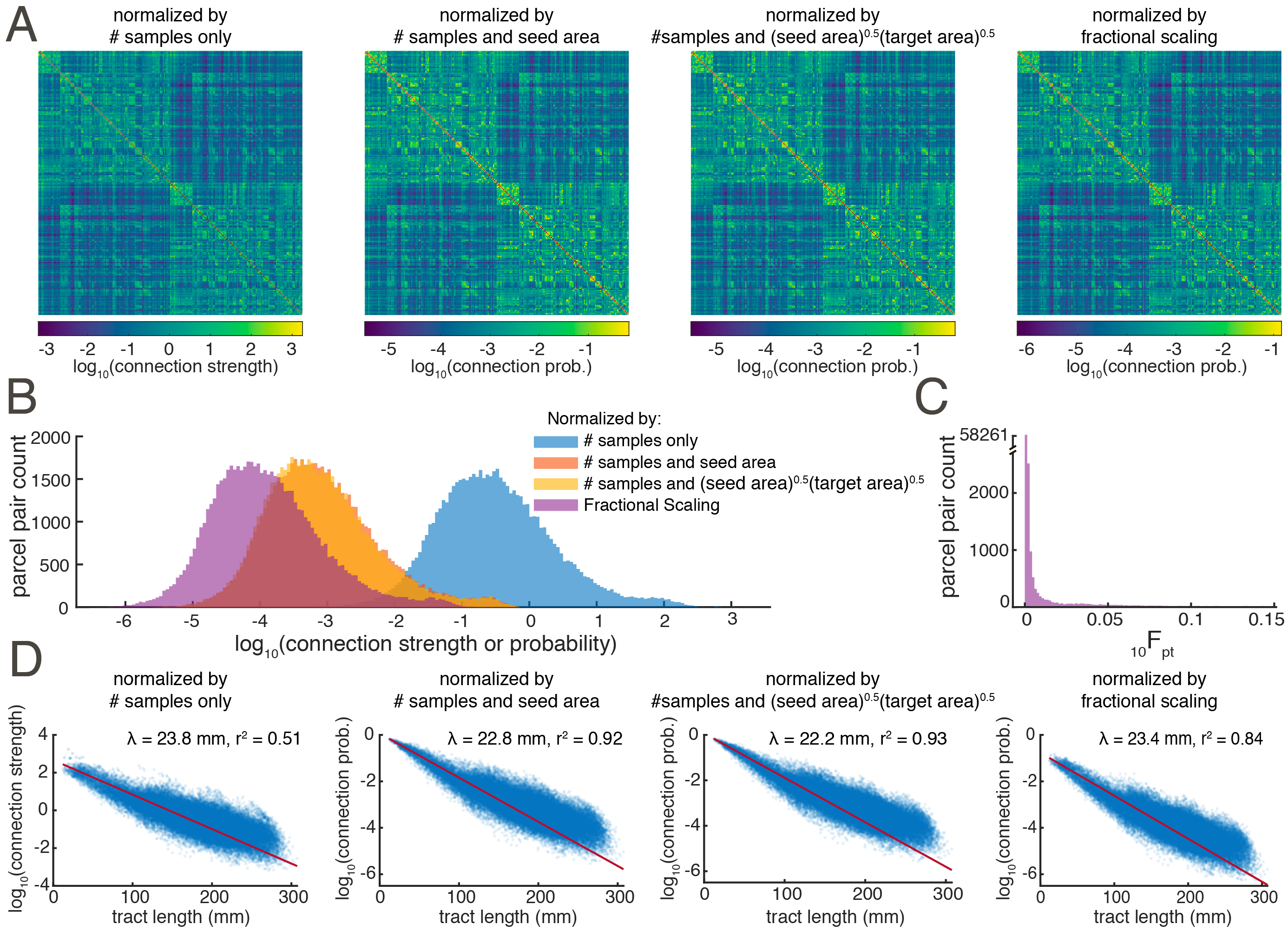

Supplement: Extended Data Figure 1-1 — Comparison of normalization methods. Shown are the (A) connectivity matrices, (B) distributions of pairwise connectivity, (C) the pre-log distribution of Fpt (D) relationships between connectivity and fiber tract length for four normalization methods. Download Figure 1-1, TIF file. [file enu-eN-NWR-0416-20-s01.tif]

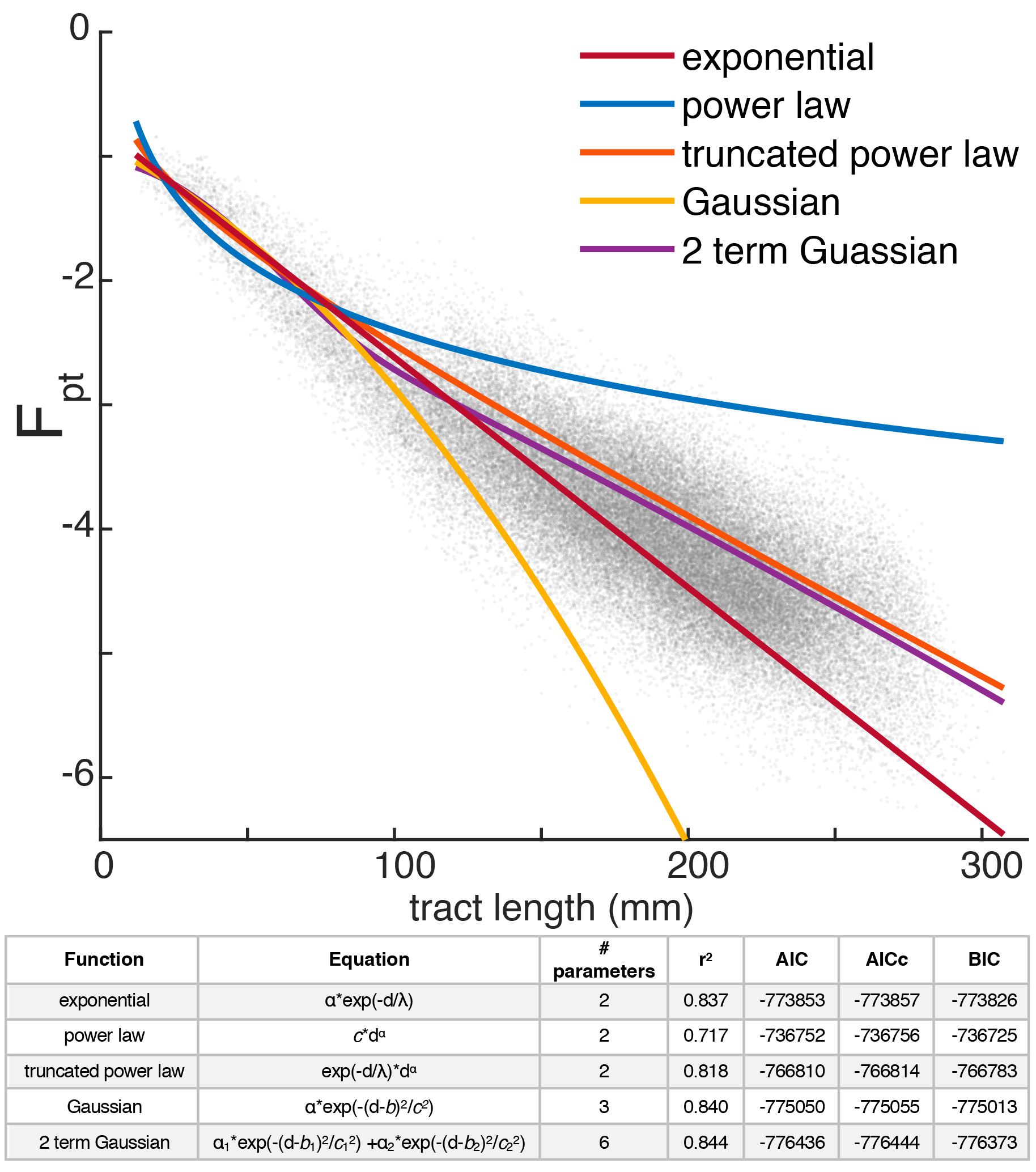

Supplement: Extended Data Figure 2-1 — Alternative models for fitting connectivity strength as a function of fiber tract length. Each gray marker shows the average pair-wise Fpt between two parcels and fiber tract length between them, as also shown in Figure 2D. The colored traces show maximum likelihood estimates for several listed functional forms. The AIC, AICc, and aBIC columns contain the Akaike, corrected Akaike, and Bayesian information criteria, respectively. While the Gaussian fits explain slightly more variance and have a slightly lower AIC than the exponential fit, the exponential has fewer parameters and is consistent with histological non-human primate evidence (Markov et al., 2013; Donahue et al., 2016; Theodoni et al., 2020). Download Figure 2-1, TIF file. [file enu-eN-NWR-0416-20-s02.tif]

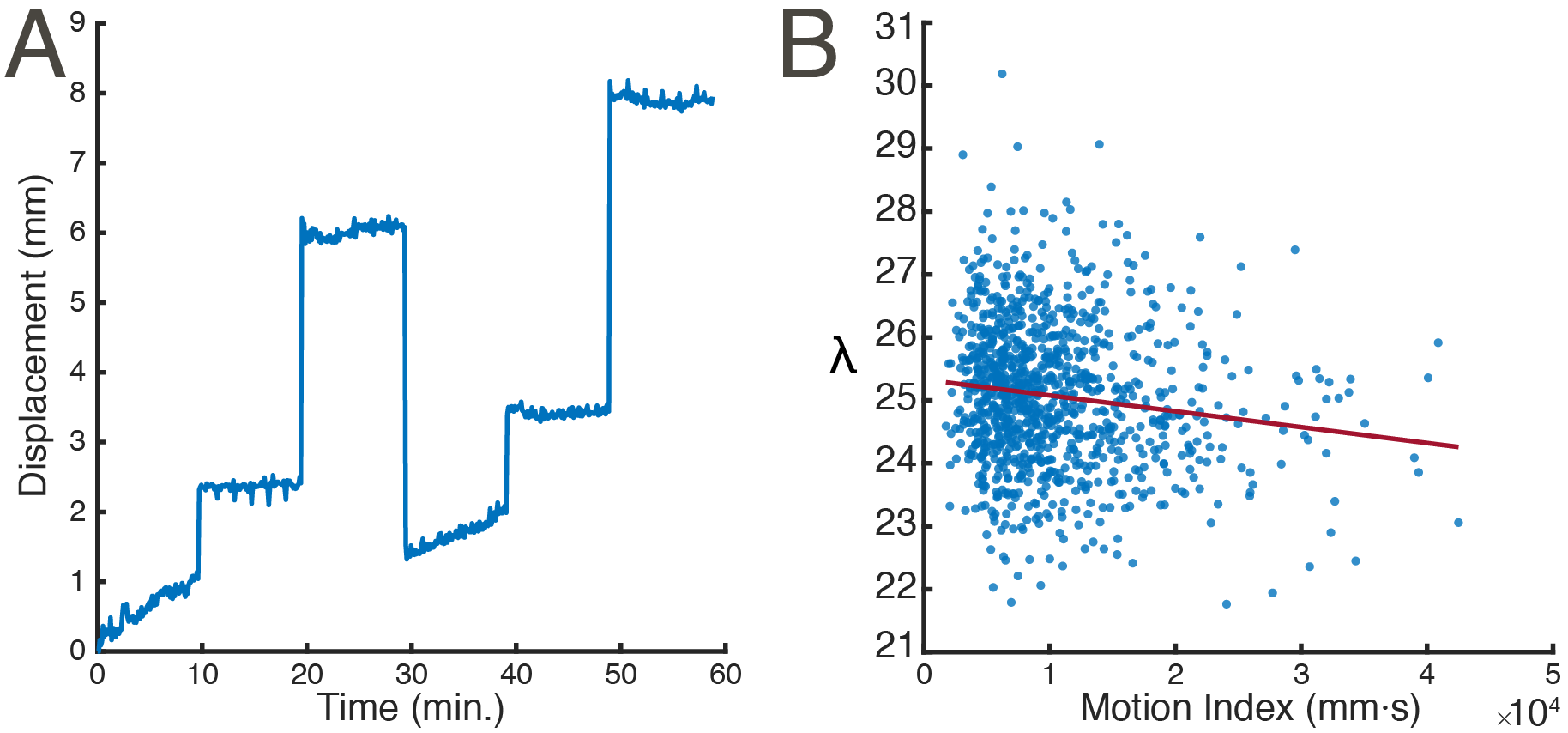

Supplement: Extended Data Figure 2-2 — Effect of motion during the dMRI scan. A, Time course of displacement relative to initial position for one subject (996782). The six runs of the HCP dMRI protocol can be seen. B, Exponential fall-off coefficient λ is only modestly affected by motion, r = 0.140, p = 4.6E-6. Each marker represents a subject. Download Figure 2-2, TIF file. [file enu-eN-NWR-0416-20-s03.tif]

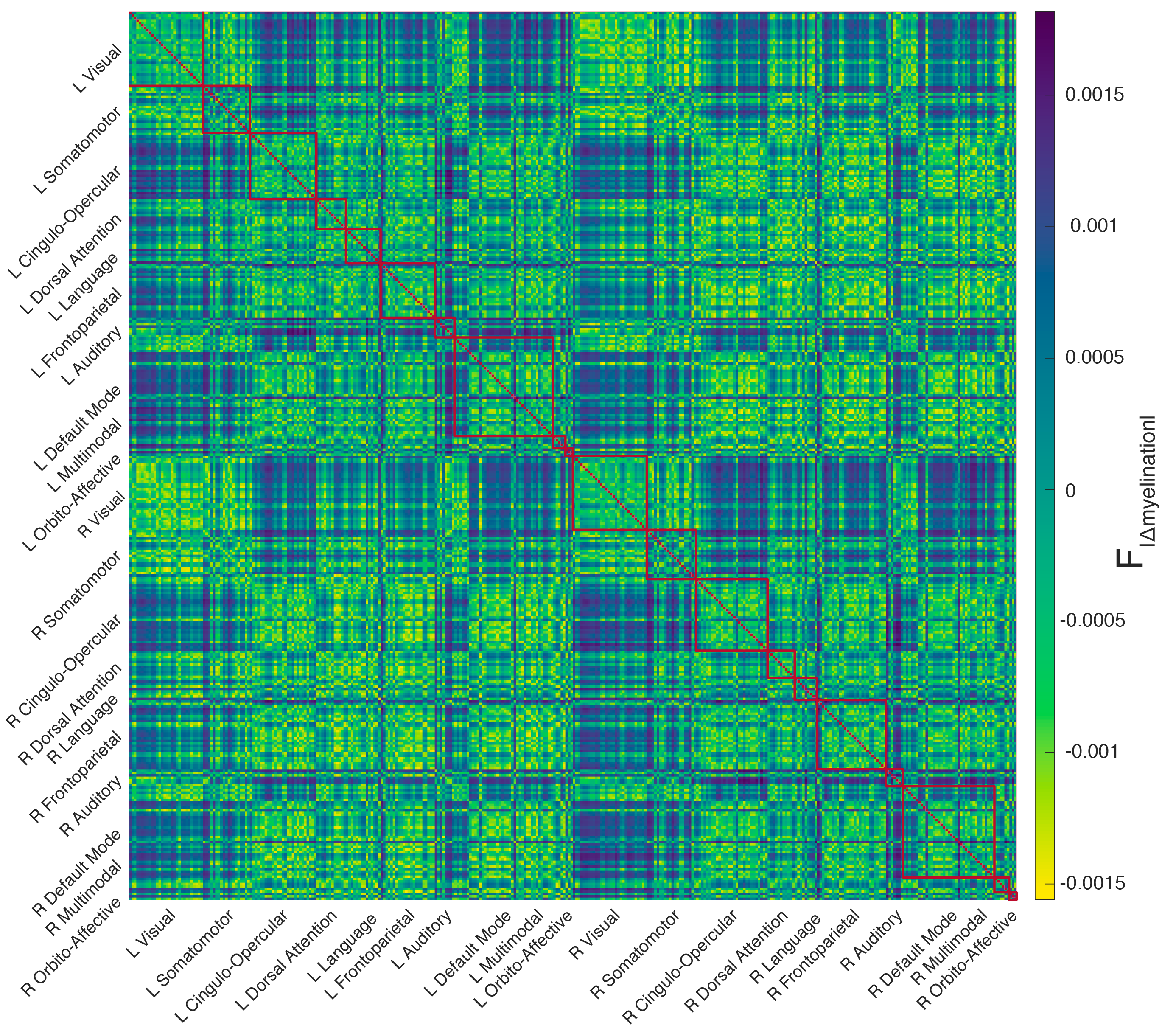

Supplement: Extended Data Figure 8-1 — Myelination difference connectivity matrix. This provides an estimate for the difference in hierarchical level between cortical parcels. Values have been fractionally scaled. Note that the color scale has been reversed when compared to Figure 1, as |Δmyelination| is inversely proportional to connectivity. Download Figure 8-1, TIF file. [file enu-eN-NWR-0416-20-s04.tif]

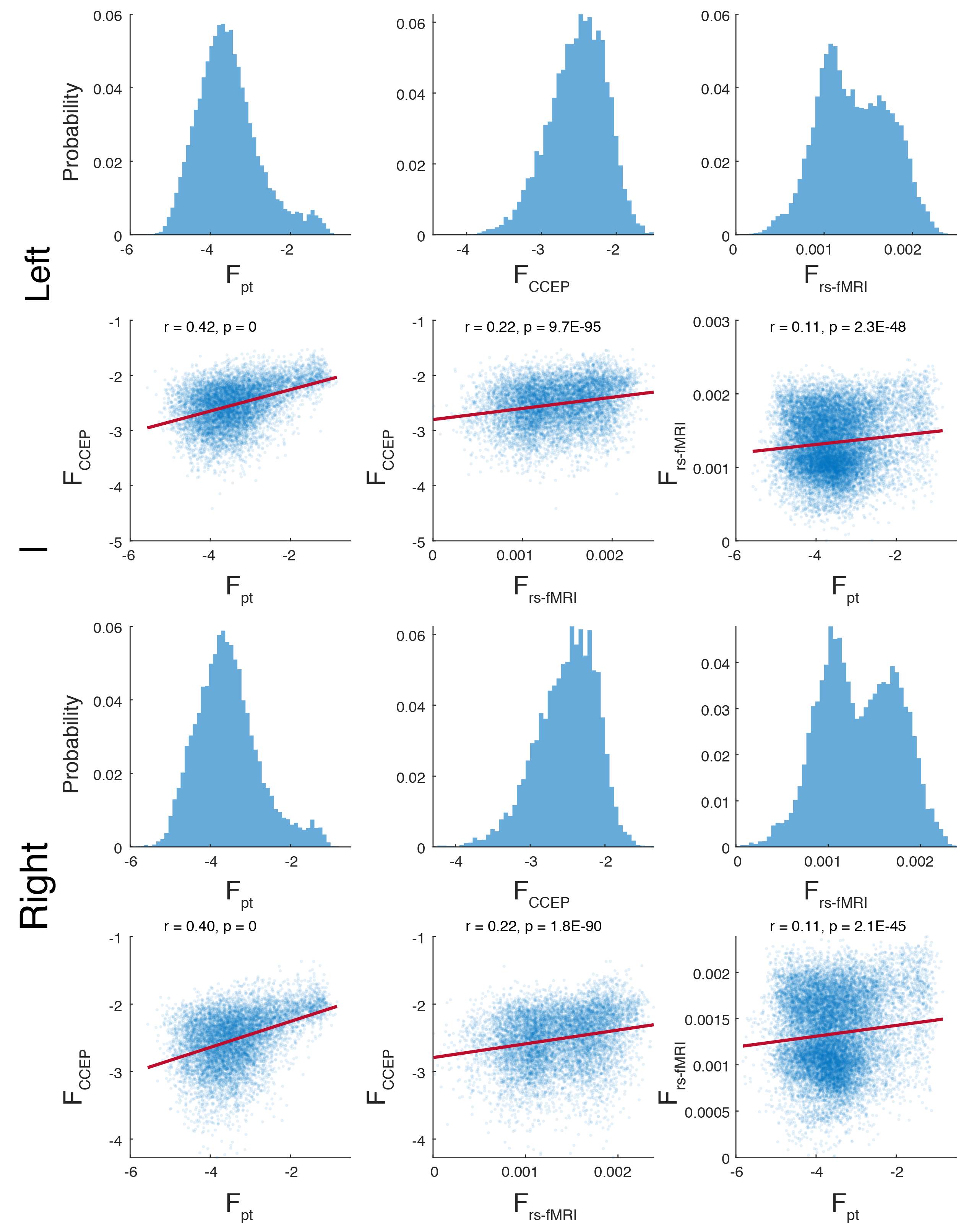

Supplement: Extended Data Figure 9-1 — Within-hemisphere comparison of probabilistic dMRI tractography, CCEP, and rs-fMRI connectivity. For the left and right hemisphere, the distribution of pairwise non-zero connection strengths and correlations among the three modalities are shown. The least-squares linear fit is shown in red. All within-hemisphere findings are concordant with the overall findings, shown in Figure 9. Download Figure 9-1, TIF file. [file enu-eN-NWR-0416-20-s09.tif]
