# Supplementary material for: A Whole-Cortex Probabilistic Diffusion Tractography Connectome
Source: eNeuro. 2021 Feb 2;8(1):ENEURO.0416-20.2020. doi: 10.1523/ENEURO.0416-20.2020 (PMC7920542; doi:10.1523/ENEURO.0416-20.2020)
Supplement: Extended Data Figure 6-1 — Differential connectivity between contralateral homologous parcels versus the mean of all other contralateral parcels. Confidence intervals are Bonferroni-corrected for multiple comparisons. Download Figure 6-1, DOCX file. [file enu-eN-NWR-0416-20-s06.docx]

| Idx | Parcel | F_pt_  to  contra-  lateral  homolog | Mean [CI_95%_]  F_pt_ to non-  homologous  contra-lateral  parcels | Idx | Parcel | F_pt_  to  contra-  lateral  homolog | Mean [CI_95%_]  F_pt_ to non-  homologous  contra-lateral  parcels | Idx | Parcel | F_pt_  to  contra-  lateral  homolog | Mean [CI_95%_]  F_pt_ to non-  homologous  contra-lateral  parcels |
| --- | --- | --- | --- | --- | --- | --- | --- | --- | --- | --- | --- |
| 1 | V1 | -1.69 | -3.72 [-3.88 -3.57] | **61** | 46 | -1.57 | -4.15 [-4.39 -3.90] | **121** | IP1 | -2.92 | -4.14 [-4.31 -3.99] |
| 2 | ProS | -4.87 | -4.71 [-4.81 -4.60] | **62** | 9-46d | -1.66 | -3.92 [-4.12 -3.70] | **122** | PFm | -3.63 | -4.09 [-4.19 -3.99] |
| 3 | DVT | -2.49 | -3.97 [-4.11 -3.83] | **63** | 43 | -1.38 | -3.65 [-3.82 -3.46] | **123** | p10p | -4.75 | -4.51 [-4.59 -4.43] |
| 4 | MST | -1.98 | -3.86 [-4.01 -3.73] | **64** | PFcm | -1.70 | -4.23 [-4.43 -3.99] | **124** | p47r | -3.80 | -4.33 [-4.46 -4.19] |
| 5 | V6 | -2.31 | -3.95 [-4.10 -3.81] | **65** | PoI2 | -1.35 | -4.09 [-4.30 -3.87] | **125** | A1 | -4.16 | -4.49 [-4.59 -4.38] |
| 6 | V2 | -3.18 | -4.25 [-4.39 -4.09] | **66** | FOP4 | -5.19 | -5.06 [-5.19 -4.91] | **126** | 52 | -4.07 | -4.37 [-4.50 -4.24] |
| 7 | V3 | -3.90 | -4.62 [-4.74 -4.49] | **67** | MI | -2.18 | -3.94 [-4.11 -3.75] | **127** | RI | -4.26 | -4.50 [-4.62 -4.38] |
| 8 | V4 | -1.66 | -3.80 [ -4.00 -3.61] | **68** | FOP1 | -3.14 | -4.45 [-4.62 -4.30] | **128** | TA2 | -4.60 | -4.62 [-4.74 -4.52] |
| 9 | V8 | -2.48 | -3.89 [-4.04 -3.72] | **69** | FOP3 | -1.38 | -3.60 [-3.78 -3.43] | **129** | PBelt | -4.84 | -4.80 [-4.92 -4.70] |
| 10 | V3A | -2.14 | -4.17 [-4.35 -4.00] | **70** | PFop | -2.06 | -3.84 [-3.99 -3.67] | **130** | MBelt | -4.68 | -4.67 [-4.77 -4.52] |
| 11 | V7 | -3.17 | -4.58 [-4.74 -4.38] | **71** | PF | -2.52 | -4.09 [-4.23 -3.93] | **131** | LBelt | -3.10 | -3.91 [-4.04 -3.80] |
| 12 | IPS1 | -2.43 | -4.14 [-4.28 -3.99] | **72** | PoI1 | -1.67 | -3.78 [-3.93 -3.62] | **132** | A4 | -4.46 | -4.47 [-4.60 -4.37] |
| 13 | FFC | -2.64 | -4.05 [-4.18 -3.89] | **73** | FOP5 | -2.67 | -4.18 [-4.33 -4.00] | **133** | 7m | -3.89 | -4.21 [-4.32 -4.10] |
| 14 | V3B | -1.88 | -3.69 [-3.83 -3.52] | **74** | PI | -2.93 | -4.17 [-4.32 -4.01] | **134** | POS1 | -3.94 | -4.29 [-4.40 -4.19] |
| 15 | LO1 | -2.11 | -3.51 [-3.64 -3.37] | **75** | a32pr | -3.53 | -4.20 [-4.31 -4.08] | **135** | 23d | -4.01 | -4.28 [-4.41 -4.17] |
| 16 | LO2 | -3.71 | -4.43 [-4.55 -4.32] | **76** | p24 | -4.24 | -4.43 [-4.56 -4.30] | **136** | v23ab | -4.25 | -4.42 [-4.52 -4.29] |
| 17 | PIT | -3.59 | -4.30 [-4.43 -4.18] | **77** | PEF | -2.49 | -4.04 [-4.17 -3.88] | **137** | d23ab | -4.26 | -4.34 [-4.44 -4.24] |
| 18 | MT | -3.83 | -4.37 [-4.49 -4.27] | **78** | 7PL | -2.85 | -4.23 [-4.37 -4.08] | **138** | 31pv | -4.08 | -4.28 [-4.38 -4.18] |
| 19 | LIPv | -4.33 | -4.60 [-4.70 -4.46] | **79** | MIP | -4.13 | -4.86 [ -5.00 -4.69] | **139** | a24 | -4.84 | -4.80 [-4.91 -4.70] |
| 20 | VIP | -3.77 | -4.56 [-4.69 -4.42] | **80** | LIPd | -4.77 | -5.06 [-5.21 -4.91] | **140** | d32 | -5.15 | -4.81 [-4.91 -4.70] |
| 21 | PH | -3.62 | -4.39 [-4.51 -4.26] | **81** | 6a | -3.03 | -4.41 [-4.57 -4.27] | **141** | p32 | -5.17 | -4.71 [-4.80 -4.61] |
| 22 | V6A | -3.72 | -4.46 [-4.59 -4.34] | **82** | PFt | -3.09 | -4.31 [-4.47 -4.16] | **142** | 10r | -2.54 | -3.96 [-4.09 -3.80] |
| 23 | VMV1 | -4.55 | -4.60 [-4.71 -4.50] | **83** | AIP | -2.38 | -4.13 [-4.31 -3.98] | **143** | 47m | -3.55 | -4.15 [-4.25 -4.03] |
| 24 | VMV3 | -4.69 | -4.75 [-4.88 -4.63] | **84** | PHA3 | -2.53 | -4.12 [-4.27 -3.95] | **144** | 8Av | -4.12 | -4.56 [-4.69 -4.44] |
| 25 | V4t | -3.63 | -4.30 [-4.42 -4.18] | **85** | TE2p | -2.68 | -4.13 [-4.29 -3.97] | **145** | 8Ad | -3.30 | -4.14 [-4.26 -4.02] |
| 26 | FST | -1.86 | -3.65 [-3.81 -3.49] | **86** | PHT | -2.64 | -4.21 [-4.34 -4.03] | **146** | 9m | -3.76 | -4.35 [-4.47 -4.24] |
| 27 | V3CD | -1.75 | -3.53 [-3.67 -3.38] | **87** | PGp | -2.30 | -3.98 [-4.12 -3.83] | **147** | 8BL | -3.46 | -4.42 [-4.54 -4.27] |
| 28 | LO3 | -4.28 | -4.52 [-4.64 -4.40] | **88** | IP0 | -1.38 | -3.89 [-4.06 -3.70] | **148** | 9p | -2.89 | -4.00 [-4.14 -3.89] |
| 29 | VMV2 | -2.00 | -3.57 [-3.68 -3.44] | **89** | 55b | -2.57 | -4.18 [-4.33 -4.06] | **149** | 10d | -3.06 | -4.00 [-4.14 -3.89] |
| 30 | VVC | -1.73 | -3.58 [-3.75 -3.41] | **90** | PSL | -2.31 | -3.99 [-4.13 -3.82] | **150** | 47l | -4.84 | -4.69 [-4.81 -4.58] |
| 31 | 4 | -2.67 | -3.96 [-4.10 -3.80] | **91** | SFL | -3.67 | -4.39 [-4.49 -4.27] | **151** | 9a | -3.42 | -4.06 [-4.16 -3.96] |
| 32 | 3b | -1.64 | -4.06 [-4.26 -3.87] | **92** | STV | -4.67 | -4.85 [-5.01 -4.72] | **152** | 10v | -2.96 | -4.13 [-4.25 -4.00] |
| 33 | 5m | -1.84 | -3.90 [-4.07 -3.72] | **93** | 44 | -1.99 | -4.15 [-4.30 -3.92] | **153** | 10pp | -3.53 | -4.67 [-4.81 -4.53] |
| 34 | 5L | -1.64 | -3.98 [-4.17 -3.77] | **94** | 45 | -4.67 | -4.70 [-4.82 -4.56] | **154** | OFC | -4.15 | -4.79 [-4.92 -4.67] |
| 35 | 24dd | -2.12 | -4.05 [-4.23 -3.81] | **95** | IFJa | -4.00 | -4.53 [-4.65 -4.42] | **155** | 47s | -4.32 | -4.61 [-4.73 -4.50] |
| 36 | 24dv | -1.94 | -3.82 [-3.98 -3.65] | **96** | IFSp | -2.78 | -4.24 [-4.41 -4.07] | **156** | EC | -4.04 | -4.49 [-4.61 -4.38] |
| 37 | 7AL | -2.49 | -3.85 [-3.98 -3.69] | **97** | STGa | -2.52 | -4.22 [-4.37 -4.04] | **157** | PreS | -4.41 | -4.44 [-4.55 -4.34] |
| 38 | 7PC | -2.47 | -3.86 [-4.04 -3.70] | **98** | A5 | -2.82 | -4.37 [-4.54 -4.21] | **158** | H | -3.82 | -4.48 [-4.60 -4.33] |
| 39 | 1 | -2.02 | -3.59 [-3.75 -3.46] | **99** | STSda | -4.16 | -4.62 [-4.76 -4.49] | **159** | PHA1 | -3.97 | -4.43 [-4.56 -4.32] |
| 40 | 2 | -1.92 | -3.90 [-4.06 -3.67] | **100** | STSdp | -4.44 | -4.65 [-4.76 -4.53] | **160** | STSvp | -4.14 | -4.75 [-4.87 -4.62] |
| 41 | 3a | -2.40 | -4.17 [-4.36 -3.97] | **101** | TPOJ1 | -4.95 | -4.80 [-4.92 -4.68] | **161** | TGd | -2.32 | -3.85 [-4.01 -3.66] |
| 42 | 6d | -2.06 | -3.50 [-3.60 -3.38] | **102** | TGv | -5.82 | -5.08 [-5.21 -4.97] | **162** | TE1a | -1.99 | -3.89 [-4.07 -3.68] |
| 43 | 6mp | -1.57 | -3.67 [-3.84 -3.42] | **103** | RSC | -4.49 | -4.43 [-4.52 -4.33] | **163** | TE2a | -3.62 | -4.48 [-4.61 -4.37] |
| 44 | 6v | -2.03 | -3.82 [-3.97 -3.66] | **104** | POS2 | -4.43 | -4.67 [-4.80 -4.54] | **164** | PGi | -1.55 | -4.22 [-4.41 -4.02] |
| 45 | OP4 | -1.97 | -3.41 [-3.55 -3.27] | **105** | 7Pm | -4.01 | -4.48 [-4.59 -4.34] | **165** | PGs | -1.51 | -4.34 [-4.55 -4.07] |
| 46 | OP1 | -2.65 | -3.76 [-3.87 -3.65] | **106** | 8BM | -4.18 | -4.21 [-4.33 -4.09] | **166** | PHA2 | -2.16 | -4.17 [-4.33 -4.02] |
| 47 | OP2-3 | -2.69 | -3.90 [-4.01 -3.78] | **107** | 8C | -4.46 | -4.46 [-4.57 -4.36] | **167** | 31pd | -4.05 | -4.16 [-4.25 -4.05] |
| 48 | FOP2 | -3.04 | -4.07 [-4.17 -3.94] | **108** | a47r | -4.41 | -4.52 [-4.67 -4.39] | **168** | 31a | -5.76 | -5.07 [-5.17 -4.94] |
| 49 | Ig | -2.55 | -3.74 [-3.85 -3.64] | **109** | IFJp | -4.91 | -4.65 [-4.77 -4.52] | **169** | 25 | -5.26 | -4.79 [-4.89 -4.66] |
| 50 | FEF | -3.14 | -4.16 [-4.26 -4.05] | **110** | IFSa | -3.04 | -4.09 [-4.23 -3.94] | **170** | s32 | -2.53 | -4.13 [-4.26 -3.97] |
| 51 | 5mv | -2.32 | -3.83 [-3.96 -3.69] | **111** | p9-46v | -5.51 | -4.88 [ -5.00 -4.76] | **171** | STSva | -2.92 | -4.24 [-4.40 -4.07] |
| 52 | 23c | -2.37 | -3.82 [ -4.00 -3.67] | **112** | a9-46v | -4.75 | -4.48 [-4.59 -4.36] | **172** | TE1m | -3.81 | -4.16 [-4.25 -4.04] |
| 53 | SCEF | -2.75 | -4.18 [-4.31 -4.03] | **113** | a10p | -4.71 | -4.76 [-4.90 -4.60] | **173** | PCV | -4.43 | -4.49 [-4.60 -4.37] |
| 54 | 6ma | -1.95 | -4.03 [-4.17 -3.88] | **114** | 11l | -5.68 | -5.03 [-5.17 -4.88] | **174** | TPOJ2 | -4.09 | -4.50 [-4.60 -4.37] |
| 55 | 7Am | -1.68 | -3.79 [-3.93 -3.64] | **115** | 13l | -6.03 | -5.14 [-5.26 -5.04] | **175** | TPOJ3 | -3.63 | -4.26 [-4.36 -4.14] |
| 56 | p24pr | -2.72 | -4.32 [-4.49 -4.13] | **116** | i6-8 | -3.63 | -4.61 [-4.74 -4.48] | **176** | PeEc | -4.70 | -4.66 [-4.77 -4.55] |
| 57 | 33pr | -1.78 | -4.08 [-4.26 -3.85] | **117** | s6-8 | -4.15 | -4.68 [-4.81 -4.56] | **177** | TF | -4.35 | -4.48 [-4.57 -4.37] |
| 58 | a24pr | -2.02 | -4.11 [-4.35 -3.87] | **118** | AVI | -3.84 | -4.27 [-4.40 -4.15] | **178** | Pir | -4.41 | -4.34 [-4.43 -4.23] |
| 59 | p32pr | -1.92 | -4.06 [-4.27 -3.84] | **119** | TE1p | -3.35 | -4.09 [-4.23 -3.96] | **179** | AAIC | -1.93 | -4.01 [-4.19 -3.82] |
| 60 | 6r | -1.97 | -3.98 [-4.21 -3.76] | **120** | IP2 | -3.10 | -4.00 [-4.11 -3.88] | **180** | pOFC | -1.76 | -4.03 [-4.25 -3.80] |

**Figure 6-1.** Differential connectivity between contralateral homologous parcels vs the mean of all other contralateral parcels. Confidence intervals are Bonferroni-corrected for multiple comparisons.
