# Supplementary material for: A Whole-Cortex Probabilistic Diffusion Tractography Connectome
Source: eNeuro. 2021 Feb 2;8(1):ENEURO.0416-20.2020. doi: 10.1523/ENEURO.0416-20.2020 (PMC7920542; doi:10.1523/ENEURO.0416-20.2020)
Supplement: Extended Data Figure 8-2 — Pearson correlations between the Fpt from each left hemisphere parcel to all others and the target parcels’ myelination indices; p values are Bonferroni-corrected for multiple comparisons. Download Figure 8-2, DOCX file. [file enu-eN-NWR-0416-20-s07.docx]

| Idx. | Parcel | r | p | Idx. | Parcel | r | p | Idx. | Parcel | r | p |
| --- | --- | --- | --- | --- | --- | --- | --- | --- | --- | --- | --- |
| 1 | V1 | 0.07 | n.s. | **61** | 46 | -0.31 | 7.18E-07 | **121** | IP1 | 0.12 | n.s. |
| 2 | ProS | 0.15 | n.s. | **62** | 9-46d | -0.31 | 4.04E-07 | **122** | PFm | 0.04 | n.s. |
| 3 | DVT | 0.23 | 3.99E-03 | **63** | 43 | 0.00 | n.s. | **123** | p10p | -0.33 | 2.77E-08 |
| 4 | MST | 0.06 | n.s. | **64** | PFcm | 0.03 | n.s. | **124** | p47r | -0.26 | 2.51E-04 |
| 5 | V6 | 0.20 | n.s. | **65** | PoI2 | -0.03 | n.s. | **125** | A1 | 0.05 | n.s. |
| 6 | V2 | 0.09 | n.s. | **66** | FOP4 | -0.14 | n.s. | **126** | 52 | 0.06 | n.s. |
| 7 | V3 | 0.12 | n.s. | **67** | MI | -0.12 | n.s. | **127** | RI | 0.01 | n.s. |
| 8 | V4 | 0.12 | n.s. | **68** | FOP1 | -0.06 | n.s. | **128** | TA2 | 0.05 | n.s. |
| 9 | V8 | 0.08 | n.s. | **69** | FOP3 | -0.10 | n.s. | **129** | PBelt | 0.10 | n.s. |
| 10 | V3A | 0.15 | n.s. | **70** | PFop | 0.05 | n.s. | **130** | MBelt | 0.08 | n.s. |
| 11 | V7 | 0.16 | n.s. | **71** | PF | 0.05 | n.s. | **131** | LBelt | 0.07 | n.s. |
| 12 | IPS1 | 0.17 | n.s. | **72** | PoI1 | -0.01 | n.s. | **132** | A4 | 0.10 | n.s. |
| 13 | FFC | 0.12 | n.s. | **73** | FOP5 | -0.10 | n.s. | **133** | 7m | -0.05 | n.s. |
| 14 | V3B | 0.16 | n.s. | **74** | PI | -0.02 | n.s. | **134** | POS1 | 0.07 | n.s. |
| 15 | LO1 | 0.15 | n.s. | **75** | a32pr | -0.30 | 2.60E-06 | **135** | 23d | -0.12 | n.s. |
| 16 | LO2 | 0.12 | n.s. | **76** | p24 | -0.27 | 6.33E-05 | **136** | v23ab | -0.08 | n.s. |
| 17 | PIT | 0.12 | n.s. | **77** | PEF | -0.16 | n.s. | **137** | d23ab | -0.10 | n.s. |
| 18 | MT | 0.05 | n.s. | **78** | 7PL | 0.13 | n.s. | **138** | 31pv | -0.13 | n.s. |
| 19 | LIPv | 0.06 | n.s. | **79** | MIP | 0.14 | n.s. | **139** | a24 | -0.31 | 1.12E-06 |
| 20 | VIP | 0.08 | n.s. | **80** | LIPd | 0.08 | n.s. | **140** | d32 | -0.34 | 1.23E-08 |
| 21 | PH | 0.10 | n.s. | **81** | 6a | -0.11 | n.s. | **141** | p32 | -0.35 | 4.09E-09 |
| 22 | V6A | 0.20 | 4.92E-02 | **82** | PFt | 0.07 | n.s. | **142** | 10r | -0.36 | 4.05E-10 |
| 23 | VMV1 | 0.09 | n.s. | **83** | AIP | 0.07 | n.s. | **143** | 47m | -0.16 | n.s. |
| 24 | VMV3 | 0.08 | n.s. | **84** | PHA3 | 0.11 | n.s. | **144** | 8Av | -0.21 | 2.27E-02 |
| 25 | V4t | 0.09 | n.s. | **85** | TE2p | 0.08 | n.s. | **145** | 8Ad | -0.30 | 3.27E-06 |
| 26 | FST | 0.08 | n.s. | **86** | PHT | 0.03 | n.s. | **146** | 9m | -0.38 | 4.11E-11 |
| 27 | V3CD | 0.17 | n.s. | **87** | PGp | 0.13 | n.s. | **147** | 8BL | -0.35 | 2.80E-09 |
| 28 | LO3 | 0.11 | n.s. | **88** | IP0 | 0.17 | n.s. | **148** | 9p | -0.33 | 4.18E-08 |
| 29 | VMV2 | 0.06 | n.s. | **89** | 55b | -0.10 | n.s. | **149** | 10d | -0.37 | 1.90E-10 |
| 30 | VVC | 0.13 | n.s. | **90** | PSL | 0.03 | n.s. | **150** | 47l | -0.15 | n.s. |
| 31 | 4 | 0.02 | n.s. | **91** | SFL | -0.29 | 1.00E-05 | **151** | 9a | -0.33 | 3.62E-08 |
| 32 | 3b | 0.04 | n.s. | **92** | STV | 0.03 | n.s. | **152** | 10v | -0.34 | 2.60E-08 |
| 33 | 5m | 0.01 | n.s. | **93** | 44 | -0.21 | 2.25E-02 | **153** | 10pp | -0.26 | 3.45E-04 |
| 34 | 5L | 0.04 | n.s. | **94** | 45 | -0.18 | n.s. | **154** | OFC | -0.28 | 3.28E-05 |
| 35 | 24dd | -0.09 | n.s. | **95** | IFJa | -0.17 | n.s. | **155** | 47s | -0.19 | n.s. |
| 36 | 24dv | -0.16 | n.s. | **96** | IFSp | -0.24 | 1.01E-03 | **156** | EC | 0.02 | n.s. |
| 37 | 7AL | 0.04 | n.s. | **97** | STGa | -0.03 | n.s. | **157** | PreS | 0.01 | n.s. |
| 38 | 7PC | 0.06 | n.s. | **98** | A5 | 0.07 | n.s. | **158** | H | 0.06 | n.s. |
| 39 | 1 | 0.06 | n.s. | **99** | STSda | 0.04 | n.s. | **159** | PHA1 | 0.07 | n.s. |
| 40 | 2 | 0.06 | n.s. | **100** | STSdp | -0.01 | n.s. | **160** | STSvp | -0.04 | n.s. |
| 41 | 3a | 0.05 | n.s. | **101** | TPOJ1 | 0.00 | n.s. | **161** | TGd | -0.11 | n.s. |
| 42 | 6d | -0.05 | n.s. | **102** | TGv | -0.03 | n.s. | **162** | TE1a | -0.04 | n.s. |
| 43 | 6mp | -0.03 | n.s. | **103** | RSC | -0.03 | n.s. | **163** | TE2a | -0.02 | n.s. |
| 44 | 6v | -0.09 | n.s. | **104** | POS2 | 0.11 | n.s. | **164** | PGi | 0.01 | n.s. |
| 45 | OP4 | 0.06 | n.s. | **105** | 7Pm | 0.00 | n.s. | **165** | PGs | 0.06 | n.s. |
| 46 | OP1 | 0.03 | n.s. | **106** | 8BM | -0.37 | 1.14E-10 | **166** | PHA2 | 0.08 | n.s. |
| 47 | OP2-3 | -0.01 | n.s. | **107** | 8C | -0.22 | 1.25E-02 | **167** | 31pd | -0.13 | n.s. |
| 48 | FOP2 | -0.06 | n.s. | **108** | a47r | -0.25 | 4.75E-04 | **168** | 31a | -0.09 | n.s. |
| 49 | Ig | -0.02 | n.s. | **109** | IFJp | -0.17 | n.s. | **169** | 25 | -0.27 | 9.02E-05 |
| 50 | FEF | -0.07 | n.s. | **110** | IFSa | -0.25 | 3.91E-04 | **170** | s32 | -0.34 | 2.28E-08 |
| 51 | 5mv | -0.07 | n.s. | **111** | p9-46v | -0.28 | 2.50E-05 | **171** | STSva | 0.02 | n.s. |
| 52 | 23c | -0.12 | n.s. | **112** | a9-46v | -0.30 | 1.33E-06 | **172** | TE1m | -0.05 | n.s. |
| 53 | SCEF | -0.20 | n.s. | **113** | a10p | -0.30 | 2.32E-06 | **173** | PCV | -0.07 | n.s. |
| 54 | 6ma | -0.13 | n.s. | **114** | 11l | -0.18 | n.s. | **174** | TPOJ2 | 0.02 | n.s. |
| 55 | 7Am | 0.01 | n.s. | **115** | 13l | -0.19 | n.s. | **175** | TPOJ3 | 0.04 | n.s. |
| 56 | p24pr | -0.18 | n.s. | **116** | i6-8 | -0.16 | n.s. | **176** | PeEc | 0.02 | n.s. |
| 57 | 33pr | -0.18 | n.s. | **117** | s6-8 | -0.27 | 5.76E-05 | **177** | TF | 0.08 | n.s. |
| 58 | a24pr | -0.26 | 2.99E-04 | **118** | AVI | -0.13 | n.s. | **178** | Pir | -0.16 | n.s. |
| 59 | p32pr | -0.26 | 1.58E-04 | **119** | TE1p | 0.01 | n.s. | **179** | AAIC | -0.18 | n.s. |
| 60 | 6r | -0.14 | n.s. | **120** | IP2 | 0.06 | n.s. | **180** | pOFC | -0.24 | 1.58E-03 |

**Figure 8-2.** Pearson correlations between the F_pt_ from each left hemisphere parcel to all others and the target parcels’ myelination indices. p values are Bonferroni-corrected for multiple comparisons.
