# Supplementary material for: A Whole-Cortex Probabilistic Diffusion Tractography Connectome
Source: eNeuro. 2021 Feb 2;8(1):ENEURO.0416-20.2020. doi: 10.1523/ENEURO.0416-20.2020 (PMC7920542; doi:10.1523/ENEURO.0416-20.2020)
Supplement: Extended Data Figure 8-3 — Pearson correlations between the Fpt from each right hemisphere parcel to all others and the target parcels’ myelination indices; p values are Bonferroni-corrected for multiple comparisons. Download Figure 8-3, DOCX file. [file enu-eN-NWR-0416-20-s08.docx]

| Idx. | Parcel | r | p | Idx. | Parcel | r | p | Idx. | Parcel | r | p |
| --- | --- | --- | --- | --- | --- | --- | --- | --- | --- | --- | --- |
| 181 | R_V1 | 0.08 | n.s. | **241** | R_46 | -0.29 | 1.20E-05 | **301** | R_IP1 | 0.13 | n.s. |
| 182 | R_ProS | 0.12 | n.s. | **242** | R_9-46d | -0.29 | 7.49E-06 | **302** | R_PFm | 0.02 | n.s. |
| 183 | R_DVT | 0.23 | 2.52E-03 | **243** | R_43 | 0.00 | n.s. | **303** | R_p10p | -0.30 | 2.07E-06 |
| 184 | R_MST | 0.11 | n.s. | **244** | R_PFcm | 0.05 | n.s. | **304** | R_p47r | -0.21 | 1.69E-02 |
| 185 | R_V6 | 0.20 | n.s. | **245** | R_PoI2 | 0.03 | n.s. | **305** | R_A1 | 0.06 | n.s. |
| 186 | R_V2 | 0.10 | n.s. | **246** | R_FOP4 | -0.12 | n.s. | **306** | R_52 | 0.12 | n.s. |
| 187 | R_V3 | 0.13 | n.s. | **247** | R_MI | -0.08 | n.s. | **307** | R_RI | 0.03 | n.s. |
| 188 | R_V4 | 0.14 | n.s. | **248** | R_FOP1 | -0.07 | n.s. | **308** | R_TA2 | 0.09 | n.s. |
| 189 | R_V8 | 0.09 | n.s. | **249** | R_FOP3 | -0.08 | n.s. | **309** | R_PBelt | 0.12 | n.s. |
| 190 | R_V3A | 0.18 | n.s. | **250** | R_PFop | 0.07 | n.s. | **310** | R_MBelt | 0.09 | n.s. |
| 191 | R_V7 | 0.19 | n.s. | **251** | R_PF | 0.05 | n.s. | **311** | R_LBelt | 0.08 | n.s. |
| 192 | R_IPS1 | 0.20 | n.s. | **252** | R_PoI1 | 0.09 | n.s. | **312** | R_A4 | 0.15 | n.s. |
| 193 | R_FFC | 0.15 | n.s. | **253** | R_FOP5 | -0.05 | n.s. | **313** | R_7m | -0.05 | n.s. |
| 194 | R_V3B | 0.18 | n.s. | **254** | R_PI | 0.08 | n.s. | **314** | R_POS1 | 0.06 | n.s. |
| 195 | R_LO1 | 0.19 | n.s. | **255** | R_a32pr | -0.29 | 6.54E-06 | **315** | R_23d | -0.10 | n.s. |
| 196 | R_LO2 | 0.17 | n.s. | **256** | R_p24 | -0.27 | 9.60E-05 | **316** | R_v23ab | -0.08 | n.s. |
| 197 | R_PIT | 0.15 | n.s. | **257** | R_PEF | -0.15 | n.s. | **317** | R_d23ab | -0.10 | n.s. |
| 198 | R_MT | 0.11 | n.s. | **258** | R_7PL | 0.16 | n.s. | **318** | R_31pv | -0.11 | n.s. |
| 199 | R_LIPv | 0.09 | n.s. | **259** | R_MIP | 0.17 | n.s. | **319** | R_a24 | -0.28 | 3.54E-05 |
| 200 | R_VIP | 0.11 | n.s. | **260** | R_LIPd | 0.08 | n.s. | **320** | R_d32 | -0.32 | 2.51E-07 |
| 201 | R_PH | 0.15 | n.s. | **261** | R_6a | -0.08 | n.s. | **321** | R_p32 | -0.35 | 5.11E-09 |
| 202 | R_V6A | 0.23 | 4.12E-03 | **262** | R_PFt | 0.07 | n.s. | **322** | R_10r | -0.37 | 1.63E-10 |
| 203 | R_VMV1 | 0.11 | n.s. | **263** | R_AIP | 0.05 | n.s. | **323** | R_47m | -0.07 | n.s. |
| 204 | R_VMV3 | 0.10 | n.s. | **264** | R_PHA3 | 0.12 | n.s. | **324** | R_8Av | -0.20 | n.s. |
| 205 | R_V4t | 0.14 | n.s. | **265** | R_TE2p | 0.12 | n.s. | **325** | R_8Ad | -0.26 | 1.58E-04 |
| 206 | R_FST | 0.14 | n.s. | **266** | R_PHT | 0.09 | n.s. | **326** | R_9m | -0.37 | 2.04E-10 |
| 207 | R_V3CD | 0.19 | n.s. | **267** | R_PGp | 0.16 | n.s. | **327** | R_8BL | -0.35 | 1.95E-09 |
| 208 | R_LO3 | 0.17 | n.s. | **268** | R_IP0 | 0.20 | n.s. | **328** | R_9p | -0.31 | 8.63E-07 |
| 209 | R_VMV2 | 0.08 | n.s. | **269** | R_55b | -0.08 | n.s. | **329** | R_10d | -0.36 | 3.48E-10 |
| 210 | R_VVC | 0.14 | n.s. | **270** | R_PSL | 0.05 | n.s. | **330** | R_47l | -0.07 | n.s. |
| 211 | R_4 | 0.04 | n.s. | **271** | R_SFL | -0.22 | 1.06E-02 | **331** | R_9a | -0.30 | 1.63E-06 |
| 212 | R_3b | 0.06 | n.s. | **272** | R_STV | 0.04 | n.s. | **332** | R_10v | -0.34 | 1.05E-08 |
| 213 | R_5m | 0.04 | n.s. | **273** | R_44 | -0.20 | n.s. | **333** | R_10pp | -0.24 | 9.97E-04 |
| 214 | R_5L | 0.06 | n.s. | **274** | R_45 | -0.12 | n.s. | **334** | R_OFC | -0.24 | 1.10E-03 |
| 215 | R_24dd | -0.06 | n.s. | **275** | R_IFJa | -0.18 | n.s. | **335** | R_47s | -0.07 | n.s. |
| 216 | R_24dv | -0.15 | n.s. | **276** | R_IFSp | -0.24 | 1.75E-03 | **336** | R_EC | 0.03 | n.s. |
| 217 | R_7AL | 0.07 | n.s. | **277** | R_STGa | 0.03 | n.s. | **337** | R_PreS | 0.01 | n.s. |
| 218 | R_7PC | 0.07 | n.s. | **278** | R_A5 | 0.11 | n.s. | **338** | R_H | 0.07 | n.s. |
| 219 | R_1 | 0.07 | n.s. | **279** | R_STSda | 0.09 | n.s. | **339** | R_PHA1 | 0.09 | n.s. |
| 220 | R_2 | 0.07 | n.s. | **280** | R_STSdp | 0.04 | n.s. | **340** | R_STSvp | 0.03 | n.s. |
| 221 | R_3a | 0.07 | n.s. | **281** | R_TPOJ1 | 0.03 | n.s. | **341** | R_TGd | -0.07 | n.s. |
| 222 | R_6d | -0.03 | n.s. | **282** | R_TGv | 0.03 | n.s. | **342** | R_TE1a | 0.01 | n.s. |
| 223 | R_6mp | -0.01 | n.s. | **283** | R_RSC | -0.05 | n.s. | **343** | R_TE2a | 0.02 | n.s. |
| 224 | R_6v | -0.08 | n.s. | **284** | R_POS2 | 0.12 | n.s. | **344** | R_PGi | 0.03 | n.s. |
| 225 | R_OP4 | 0.07 | n.s. | **285** | R_7Pm | 0.03 | n.s. | **345** | R_PGs | 0.08 | n.s. |
| 226 | R_OP1 | 0.04 | n.s. | **286** | R_8BM | -0.35 | 1.71E-09 | **346** | R_PHA2 | 0.08 | n.s. |
| 227 | R_OP2-3 | 0.00 | n.s. | **287** | R_8C | -0.21 | 1.51E-02 | **347** | R_31pd | -0.12 | n.s. |
| 228 | R_FOP2 | -0.05 | n.s. | **288** | R_a47r | -0.23 | 3.49E-03 | **348** | R_31a | -0.09 | n.s. |
| 229 | R_Ig | 0.00 | n.s. | **289** | R_IFJp | -0.15 | n.s. | **349** | R_25 | -0.24 | 1.61E-03 |
| 230 | R_FEF | -0.05 | n.s. | **290** | R_IFSa | -0.21 | 3.22E-02 | **350** | R_s32 | -0.32 | 1.37E-07 |
| 231 | R_5mv | -0.04 | n.s. | **291** | R_p9-46v | -0.27 | 8.34E-05 | **351** | R_STSva | 0.08 | n.s. |
| 232 | R_23c | -0.09 | n.s. | **292** | R_a9-46v | -0.28 | 2.20E-05 | **352** | R_TE1m | 0.02 | n.s. |
| 233 | R_SCEF | -0.16 | n.s. | **293** | R_a10p | -0.25 | 8.16E-04 | **353** | R_PCV | -0.05 | n.s. |
| 234 | R_6ma | -0.08 | n.s. | **294** | R_11l | -0.13 | n.s. | **354** | R_TPOJ2 | 0.06 | n.s. |
| 235 | R_7Am | 0.04 | n.s. | **295** | R_13l | -0.11 | n.s. | **355** | R_TPOJ3 | 0.08 | n.s. |
| 236 | R_p24pr | -0.17 | n.s. | **296** | R_i6-8 | -0.13 | n.s. | **356** | R_PeEc | 0.04 | n.s. |
| 237 | R_33pr | -0.18 | n.s. | **297** | R_s6-8 | -0.22 | 9.03E-03 | **357** | R_TF | 0.13 | n.s. |
| 238 | R_a24pr | -0.26 | 2.39E-04 | **298** | R_AVI | -0.03 | n.s. | **358** | R_Pir | -0.11 | n.s. |
| 239 | R_p32pr | -0.25 | 4.93E-04 | **299** | R_TE1p | 0.07 | n.s. | **359** | R_AAIC | -0.09 | n.s. |
| 240 | R_6r | -0.14 | n.s. | **300** | R_IP2 | 0.03 | n.s. | **360** | R_pOFC | -0.20 | 3.99E-02 |

**Figure 8-3.** Pearson correlations between the F_pt_ from each right hemisphere parcel to all others and the target parcels’ myelination indices. p values are Bonferroni-corrected for multiple comparisons.
